# *CITED2* and *NCOR2* in anti-oestrogen resistance and progression of breast cancer

**DOI:** 10.1038/sj.bjc.6605423

**Published:** 2009-11-10

**Authors:** T van Agthoven, A M Sieuwerts, J Veldscholte, M E Meijer-van Gelder, M Smid, A Brinkman, A T den Dekker, I M Leroy, W F J van IJcken, S Sleijfer, J A Foekens, L C J Dorssers

**Affiliations:** 1 Department of Pathology, Erasmus MC – University Medical Center Rotterdam, Rotterdam 3000 CA, The Netherlands; 2 Department of Medical Oncology, Erasmus MC – University Medical Center Rotterdam, Rotterdam 3000 CA, The Netherlands; 3 Cancer Genomics Center, Erasmus MC – University Medical Center Rotterdam, Rotterdam 3000 CA, The Netherlands; 4 Erasmus Center of Biomics, Erasmus MC – University Medical Center Rotterdam, Rotterdam 3000 CA, The Netherlands

**Keywords:** tamoxifen, drug resistance, functional screen, insertion mutagenesis, gene expression profile, prognostic and predictive markers

## Abstract

**Background:**

Endocrine therapies of breast cancer are effective but ultimately fail because of the development of treatment resistance. We have previously revealed several genes leading to tamoxifen resistance *in vitro* by retroviral insertion mutagenesis. To understand the manner in which these genes yield tamoxifen resistance, their effects on global gene expression were studied and those genes resulting in a distinct gene expression profile were further investigated for their clinical relevance.

**Methods:**

Gene expression profiles of 69 human breast cancer cell lines that were made tamoxifen resistant through retroviral insertion mutagenesis were obtained using oligonucleotide arrays and analysed with bioinformatic tools. mRNA levels of *NCOR2* and *CITED2* in oestrogen receptor-positive breast tumours were determined by quantitative RT–PCR. mRNA levels were evaluated for association with metastasis-free survival (MFS) in 620 patients with lymph node-negative primary breast cancer who did not receive systemic adjuvant therapy, and with clinical benefit in 296 patients receiving tamoxifen therapy for recurrent breast cancer.

**Results:**

mRNA expression profiles of most tamoxifen-resistant cell lines were strikingly similar, except for the subgroups of cell lines in which *NCOR2* or *CITED2* were targeted by the retrovirus. Both *NCOR2* and *CITED2* mRNA levels were associated with MFS, that is, tumour aggressiveness, independently of traditional prognostic factors. In addition, high *CITED2* mRNA levels were predictive for a clinical benefit from first-line tamoxifen treatment in patients with advanced disease.

**Conclusions:**

Most retrovirally targeted genes yielding tamoxifen resistance in our cell lines do not impose a distinctive expression profile, suggesting that their causative role in cell growth may be accomplished by post-transcriptional processes. The associations of *NCOR2* and *CITED2* with outcome in oestrogen receptor-positive breast cancer patients underscore the clinical relevance of functional genetic screens to better understand disease progression, which may ultimately lead to the development of improved treatment options.

Tamoxifen is widely applied in the treatment of breast cancer. Its efficacy has been established in both the adjuvant setting to reduce recurrences in localised disease (Early Breast Cancer Trialists’ Collaborative Group, 2005) and in the palliative management of patients with advanced disease (Jaiyesimi *et al*, 1995; Jordan, 1995; Osborne, 1998). In agreement with its biological function as an anti-oestrogen, tamoxifen requires the presence of the oestrogen receptor (ER) alpha. But a substantial number of ER*α*-positive breast cancer patients will not benefit from tamoxifen treatment because of intrinsic or *de novo* resistance. Furthermore, nearly all responsive patients will experience disease progression because of the development of acquired resistance. Although other anti-oestrogens and aromatase inhibitors have been developed, resistance to these compounds will occur (Nabholtz *et al*, 2000; Mouridsen *et al*, 2003; Paridaens *et al*, 2003; Howell, 2006). Despite intense research in the last few decades into the failure of endocrine therapies (Ali and Coombes, 2002; Clarke *et al*, 2003; Jansen *et al*, 2005; Osborne *et al*, 2005; Riggins *et al*, 2007; Creighton *et al*, 2008; Hurtado *et al*, 2008; Iorns *et al*, 2008), more insight into the underlying mechanisms is definitely needed.

Previously, we executed a non-biased functional genetic screen in an oestrogen-dependent human breast cancer cell model, aimed at identifying genes causing tamoxifen resistance. Insertion of a defective retrovirus into the genome randomly introduced individual genetic changes in ZR-75-1 cells (Dorssers *et al*, 1993). Infected cells were then selected for their ability to proliferate while being exposed to tamoxifen. From these cultures, 79 stable cell lines were established. Using various molecular strategies, common virus insertion sites (cVIS) were mapped, enabling the identification of candidate genes that cause the resistant phenotype. These retroviral targets were collectively termed breast cancer anti-oestrogen-resistance (*BCAR*) genes. Ultimate proof for the role of seven target genes (*AKT1*, *AKT2*, *BCAR1*, *BCAR3*, *EGFR*, *GRB7* and *TRERF1*) was obtained by cDNA transfection into oestrogen-dependent cancer cells, transforming them into a tamoxifen-resistant phenotype (Van Agthoven *et al*, 1998, 2009b; Brinkman *et al*, 2000).

To elucidate the manner in which these retroviral target genes induce tamoxifen resistance in ZR-75-1 cells, we assessed whether differences in gene expression patterns existed between the different groups of tamoxifen-resistant cell lines. Two candidate target genes, *CITED2* and *NCOR2*, yielding distinct gene expression profiles when compared with other cell lines, were analysed for their clinical relevance in terms of tamoxifen resistance and tumour aggressiveness.

## Materials and methods

### Cell culture

The panel of tamoxifen-resistant cell lines was previously generated by retrovirus infection and selection for resistance to 4-hydroxy-tamoxifen (OH-Tam, Sigma-Aldrich Chemie BV, Zwijndrecht, The Netherlands) (Dorssers *et al*, 1993). Cell lines were cultured in RPMI supplemented with 15% bovine calf serum, 10% CRIP supernatant and 1 *μ*M of OH-Tam as described before (Dorssers *et al*, 1993).

### Gene expression profiling

Total RNA was prepared by direct lysis with RNA-Bee (Bio-connect, Huissen, The Netherlands) of approximately 80% confluent cells. cDNA synthesis was performed using an T7dT-oligo primer, 3 *μ*g of total RNA and reverse transcriptase (Superscript II, Invitrogen, Breda, The Netherlands). Second-strand synthesis was carried out by *E.coli* ligase, *E.coli* DNA polymerase (Invitrogen) and RNase H (Promega Benelux b.v., Leiden, The Netherlands). The double-stranded cDNA was purified on Quiaquick PCR columns (Qiagen, Hilden, Germany). *In vitro* transcription using the T7 Megascript Kit (Ambion, Austin, TX, USA) was used to produce amplified RNA (aRNA). Further details are presented in the Supplementary information.

Spotted oligo microarrays with the Operon V3.0 library (35K Human, http://omad.operon.com/humanV3) were obtained from the Netherlands Cancer Institute Central Microarray Facility (NKI-CMF). Protocols for sample preparation were taken from the NKI-CMF website (http://microarrays.nki.nl) and are detailed elsewhere (Meester-Smoor *et al*, 2008). In short, 1 *μ*g of amplified RNA was labelled using the ULS-Cy3/5 aRNA fluorescent labelling kit (Kreatech, Amsterdam, The Netherlands), and was used for hybridisation (Supplementary information). Two independent samples of a cell line were hybridised against the reference and one of them was also used in a dye-swap hybridisation. Hybridised arrays were scanned on a ScanArray Express HT instrument (Perkin Elmer Life and Analytical Sciences BV, Groningen, The Netherlands). Fluorescence intensities were determined using ImaGene software version 6.0 (Biodiscovery, El Segundo, CA, USA), uploaded into the CMF database (CMFdb, http://cmfdb.nki.nl) and normalised using the lowess subarray method. Both raw and normalised data have been deposited in NCBI's Gene Expression Omnibus (Edgar *et al*, 2002) and are accessible through GEO Series accession number GSE14513 (http://www.ncbi.nlm.nih.gov/geo/query/ acc.cgi?acc=GSE14513).

Analyses on normalised data were performed using BRB-ArrayTools developed by Dr Richard Simon and the BRB-ArrayTools Development Team (http://linus.nci.nih.gov/BRB-ArrayTools.html). Supervised class comparisons (Supplementary information and Supplementary Table S1) were performed to select genes with differential expression in cell lines grouped according to the presence of the retrovirus in a particular cVIS (Van Agthoven *et al*, 2009b). Hierarchical clustering was carried out with Spotfire DecisionSite 9.0 (Tibco, Somerville, MA, USA). To analyse selected genes in human breast cancer microarray data (Wang *et al*, 2005), spots were linked to Affymetrix probe sets using Entrez Gene and Ensemble identifiers (Supplementary Table S2).

### Patient samples

The protocol to study biological markers associated with disease outcome was approved by the medical ethics committee of the Erasmus Medical Center Rotterdam, The Netherlands (MEC 02.953). This retrospective study used 791 blindly coded, ER*α* protein-positive (⩾10 fmol/mg of protein) primary tumour tissues, in accordance with the Code of Conduct of the Federation of Medical Scientific Societies in the Netherlands (http://www.fmvv.nl). This report is as much as possible in line with the REMARK guidelines (McShane *et al*, 2006). Primary breast tumours were obtained from patients with detailed clinical follow-up as previously described (Sieuwerts *et al*, 2006, 2007; Jansen *et al*, 2007; Meijer *et al*, 2008, 2009; Van Agthoven *et al*, 2009a). All patients underwent breast surgery for breast cancer from 1978 through 2000. ER*α* status was determined by routine ligand-binding assays or by enzyme immunoassays (Foekens *et al*, 1989). Further patient characteristics are summarised in Supplementary Table S3.

To evaluate the impact of individual genes on tamoxifen resistance, 296 patients who received tamoxifen monotherapy as first-line treatment for advanced disease were included (Sieuwerts *et al*, 2005). Of these patients, 10% presented with distant metastasis at diagnosis (Supplementary Table S3). None of these patients (43% node negative) had received prior adjuvant hormonal therapy, whereas 56 patients had received prior adjuvant chemotherapy (22 patients anthracycline based (FAC/FEC), 34 patients non-anthracycline based (CMF)). All patients were routinely followed up as previously described (Martens *et al*, 2005; Meijer *et al*, 2008). The type of response to tamoxifen therapy was assessed according to standard criteria (Hayward *et al*, 1977; EORTC Breast Cancer Cooperative Group, 2000). Clinical benefit, defined as objective tumour response or no change lasting longer than 6 months, was observed in 185 patients (62.5%); 13 achieved complete remission, 38 partial remission and the remaining 134 patients experienced no change lasting longer than 6 months. A total of 111 patients did not experience clinical benefit (94 progressive disease and 17 no change less than or equal to 6 months). The median time to progression was 8.5 months. The median follow-up time after start of therapy of patients alive (*n*=78) was 38.7 months.

For the analysis of the association of individual genes with tumour aggressiveness, 620 lymph node-negative (LNN) patients with ER*α* protein-positive tumours were included (Supplementary Table S3). Of these patients, 52% had undergone breast-conserving lumpectomy and 100% node dissection. Adjuvant radiotherapy was administered to 58% of the patients, none of whom had received adjuvant systemic therapy. Distant recurrences were observed in 215 patients (34.7%), and the median follow-up for patients alive (*n*=427) was 93.4 months. A total of 193 deaths were recorded.

### Quantitative reverse transcription (RT)–PCR of breast tumours

RNA isolation, cDNA synthesis, quality control checks and normalisation of data on a set of three reference genes (*HPRT1*, *HBMS* and *B2M*) were carried out as previously described (Sieuwerts *et al*, 2005; Meijer *et al*, 2008). Real-time quantitative RT–PCR was performed using the Stratagene Mx3000P QPCR System (Agilent Technologies, Waldbronn, Germany), Assay-on-Demand kits for *CITED2* (Hs00366696_m1) and *NCOR2* (Hs00196955_m1) from Applied Biosystems (Nieuwerkerk a/d IJssel, The Netherlands), and the ABsolute qPCR Low ROX master Mix from Abgene Ltd (Epsom, UK). Quantification of *ESR1*, *PGR* and *MKI67* mRNA levels was performed as described (Sieuwerts *et al*, 2005, 2007).

### Statistical analyses

For statistical computations, STATA statistical package 10.0 (STATA Corp., College Station, TX, USA) was used. Differences in levels between groups were assessed using the Mann–Whitney *U-*test or the Kruskal–Wallis test, including a Wilcoxon-type test for trend, when appropriate. In these tests, patient and tumour characteristics were used as grouping variables. The strength of the association between continuous variables was tested with Spearman's rank correlation (r_s_). To reduce skewness, most variables were log- or Box-Cox transformed. The Cox proportional hazards model was used to calculate the hazard ratio (HR) and its 95% confidence interval (CI) in the analyses of metastasis-free survival (MFS), overall survival (OS) and progression-free survival (PFS). MFS was defined as the time between surgical removal of the primary tumour and the first detection of a distant metastasis, as revealed after symptoms were reported by the patient, the occurrence of clinical signs or at regular follow-up. Death from any cause was considered an event for OS. For advanced patients treated with tamoxifen, PFS was defined as the time elapsed between initiation of tamoxifen therapy and the first detection of progression of the disease. The proportional hazards assumption was not violated for MFS, as verified by a test based on Schoenfeld residuals. The proportional hazards assumption was violated for PFS, but not when follow-up was censored at 9 months as described previously (Sieuwerts *et al*, 2005). Logistic regression analysis was used to examine the relation of mRNA levels with clinical benefit of tamoxifen therapy, and for the calculation of the Odds Ratio (OR) and its 95% CI. A two-sided *P*-value of <0.05 was considered to be statistically significant.

## Results

### Gene expression profiles of tamoxifen-resistant cell lines are very similar

As all tamoxifen-resistant cell lines generated by insertion mutagenesis were derived from a single parental cell line (Dorssers *et al*, 1993; Van Agthoven *et al*, 2009b), it was hypothesised that differences in gene expression profiles would reflect the underlying mechanisms by which the retrovirally targeted genes induced tamoxifen resistance. From a total of 69 out of 79 cell lines, high-quality total RNA from two independent cell cultures was converted into double-stranded cDNA and amplified into aRNA with T7-RNA polymerase. Fluorophore-labelled probes of tamoxifen-resistant cell lines were hybridised onto a 35K oligonucleotide array containing approximately 28 000 annotated human genes, and compared with a reference probe derived from a panel of stable cell lines (Jansen *et al*, 2005). Our tamoxifen-resistant cell line panel showed clearly different gene expression patterns when compared with oestrogen-stimulated or tamoxifen-arrested parental ZR-75-1 cells (not shown).

Among the tamoxifen-resistant cell lines, unsupervised hierarchical clustering revealed very similar expression profiles (Supplementary Figure S1, 15 376 genes) and did not group the bulk of cell lines belonging to a particular cVIS (Van Agthoven *et al*, 2009b). Only cell lines with a viral integration in *NCOR2* were mostly positioned in close proximity within the dendrogram (Supplementary Figure S1, not indicated).

**Figure 1:**
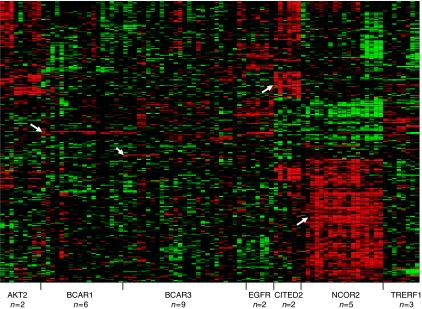
Hierarchical clustering of 251 genes in tamoxifen-resistant breast cancer cell lines. Cell lines (three hybridisations each) were grouped together according to the location of the retrovirus in the cellular genome (columns). The number of different cell lines in a group is indicated below the name. Genes (rows) were selected by class comparison analysis and clustering was performed using the unweighted pair-group method with arithmetic mean in Spotfire. Gene expression above the mean is shown in white, that below the mean is indicated in black. Target genes in the integration loci are marked with arrows (from left to right: *BCAR1*, *BCAR3*, *CITED2* and *NCOR2*). The order of the genes, their names and identifiers are provided in Supplementary Table S1.

Subsequently, a class comparison analysis in which cell lines were organised according to the presence of a retrovirus in the same chromosomal region (Van Agthoven *et al*, 2009b) was performed. Between cell lines with an integration near *BCAR1* or *BCAR3,* very few differences were observed, in accordance with the results of our previous analysis of *BCAR1*- or *BCAR3*-transfected cells (Dorssers *et al*, 2005). Therefore, these cell lines were combined and used as the reference group for class comparison with each of the other groups of cell lines. All genes showing significant differences (*P*<0.001; *N*=1106, Supplementary Table S1) were pooled. After excluding genes showing a dye bias, 251 spots (representing 194 unique annotated genes) were left for hierarchical clustering analysis. The cell lines were organised into groups having a retroviral integration in the same chromosomal region (Figure 1 and Supplementary Figure S2). Cell lines belonging to the NCOR2 group showed the most prominent differences, having a higher expression of many genes, compared with the other tamoxifen-resistant cell lines. The retroviral target gene in the integration locus (*NCOR2*) was robustly upregulated in these cell lines (Figure 1, most right arrow). In agreement with the increased expression, western blot analysis confirmed that NCOR2 protein levels were increased in these cell lines (Supplementary Figure S3). The cell lines with an integration in the *CITED2* locus also showed an altered expression of several genes, including strongly increased levels of the targeted *CITED2* gene (Figure 1). This is in agreement with the previously established overexpression of *CITED2* mRNA and protein in these cell lines (Van Agthoven *et al*, 2009b). Individual cell lines with a retroviral integration near *AKT2* or *TRERF1* showed different expression patterns, suggesting clonal variation (Figure 1). Cell lines with integrations within cVIS5, 7, 10 or 11 (for which the responsible target genes have not yet been established (Van Agthoven *et al*, 2009b)) and cell lines lacking a common virus integration site did not show strongly different expression patterns (Supplementary Figure S2). Our class comparison analyses revealed a novel target gene (*TSHZ1*) in the cVIS6 locus, which was sevenfold upregulated as a consequence of virus integration (Supplementary Figure S2, arrow), but in this cell line, global gene expression was only minimally changed.

From these results, we conclude that in many cell lines, the gene causative for oestrogen independency did not impose a distinct and permanent expression phenotype. Only the virus integrations near *NCOR2* and *CITED2* caused a shift in gene expression.

### NCOR2 gene signature correlates with molecular subtypes in ER-positive breast cancer patients

To analyse the clinical relevance of 171 genes differentially expressed in cell lines with a retroviral integration in *NCOR2*, compared with cell lines with an integration in *BCAR1* and *BCAR3* (Supplementary Table S2), we performed hierarchical clustering of the expression data from our series of 221 ER-positive breast tumours of LNN patients who did not receive adjuvant systemic therapy (Wang *et al*, 2005). Surprisingly, hierarchical clustering (Supplementary Figure S4a, Supplementary Information) of these genes resulted in a sample dendrogram, which showed a strong correlation with the molecular subtypes of these tumours (Perou *et al*, 2000; Smid *et al*, 2008). A detailed inspection showed that one branch consisted of tumours belonging to the normal-like and ERBB2 subtypes and nearly half of the tumours of luminal A-type (group A), whereas the two other groups contained tumours predominantly belonging to the luminal A- or B subtype (groups B and C). Patients with tumours belonging to cluster group A showed a prolonged MFS when compared with the MFS of the other two groups of patients (Supplementary Figure S4b).

### Role of *CITED2* and *NCOR2* in breast cancer aggressiveness and tamoxifen resistance

Given the impact of the viral integrations within *NCOR2* and *CITED2* on the overall mRNA expression in our cell model, we established their relationships with clinical parameters reflecting tamoxifen resistance and tumour aggressiveness. The mRNA levels of these two genes were determined in primary ER*α*-positive tumours by quantitative RT–PCR and normalised to three reference genes. A correlation was observed between *NCOR2* and *CITED2*, *NCOR2* and *MKI67*, and between *CITED2* and *ESR1* (*r*_s_>0.20, *P*<0.0001) (Supplementary Table S4). Increasing *CITED2* mRNA levels were correlated with older age and post-menopausal status. Lower *NCOR2* levels correlated with a higher grade (Supplementary Table S4).

For the analysis of association with tamoxifen treatment, 296 patients who received tamoxifen as first-line treatment for advanced disease (Supplementary Table S3) were included. In the univariate logistic regression analysis, high *CITED2* mRNA levels were significantly associated with clinical benefit, both as a continuous (OR=1.34, *P*=0.028, Table 1) and as a categorised variable (median, OR=2.20, *P*=0.001). In multivariate analyses including the traditional predictive factors (that is, age and menopausal status at the start of tamoxifen therapy, disease-free interval, dominant site of relapse and *ESR1* and *PGR* mRNA levels), high *CITED2* levels remained significantly associated with clinical benefit (Table 1). Continuous *CITED2* mRNA levels were of borderline significance for PFS. When divided into two equally sized groups, high levels of *CITED2* mRNA were associated with favourable PFS, independently of traditional predictive factors (Table 1). In contrast, *NCOR2* mRNA levels as a continuous or as a categorised variable were not significantly associated with clinical benefit or PFS (Table 1). The results of the multivariate analyses with adjuvant chemotherapy as an additional variable showed that the estimates of the coefficients of individual genes were similar.

**Figure 2:**
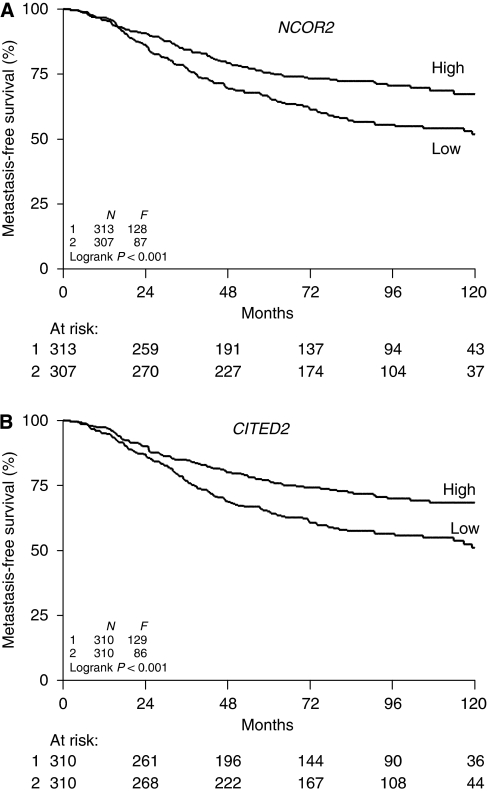
Metastasis-free-survival of 620 lymph node-negative patients with ER*α*-positive breast cancer. Kaplan–Meier curves for MFS for subgroups of patients as a function of the *NCOR2* (**A**) or *CITED2* (**B**) mRNA levels of primary tumours. Patients were divided into two groups having primary tumours with high (above median) or low mRNA levels. The *y* axis shows the percentage of patients without distant metastasis. Patients at risk (i.e., without event and not censored) at 24-month intervals are indicated. *N*, number of patients; *F*, number of patients with distant recurrences.

For the analysis of tumour aggressiveness, 620 ER*α*-positive tumours of LNN patients who did not receive any systemic adjuvant treatment were included (Supplementary Table S3). In the analysis of MFS and OS, high levels of *NCOR2* mRNA (both as a continuous and categorised variable, Table 2) were associated with a favourable outcome. The Kaplan–Meier analysis visualises the different outcomes of patients stratified according to the median level of *NCOR2* (Figure 2A). In a multivariate analysis including traditional prognostic factors (age, menopausal status, tumour size, grade and *ESR1* and *PGR* mRNA levels), *NCOR2* mRNA levels were significantly associated with a favourable MFS (median, HR=0.68, *P*=0.006) and OS (Table 2). The analysis of *CITED2* showed similar results. High levels of *CITED2,* both as a continuous and categorised variable, were significantly associated with prolonged MFS (Table 2). Kaplan–Meier curves illustrate the different outcomes for patients stratified according to the median *CITED2* mRNA level (Figure 2B). These associations with MFS were independent of the classical prognostic factors (HR=0.71, *P*=0.017, Table 2).

## Discussion

Insertion mutagenesis with retroviruses is a powerful tool to identify genes involved in sensitivity to anti-cancer agents. With this approach, we have been able to identify several genes conferring tamoxifen resistance *in vitro* (Van Agthoven *et al*, 1998, 2009b; Brinkman *et al*, 2000). To obtain a better insight into the mechanisms by which these genes induce tamoxifen resistance, we assessed whether differences in gene expression patterns existed between the different groups of tamoxifen-resistant cell lines. We found that most groups of cell lines showed a very similar pattern of gene expression, but that cell lines with a retroviral integration near *NCOR2* or *CITED2* showed a different gene expression profile. NCOR2 and CITED2 both function as regulators of transcription and therefore altered gene expression patterns could be expected by their deregulation. In contrast, other nuclear regulators (such as TRERF1), did not significantly change the expression profile. Importantly, for some groups of cell lines (for example, those with insertions near the *AKT2* gene), differences in gene expression patterns were observed among the independently derived cell lines, suggesting clonal variations not related to the specific target gene. These observations imply that the changes in gene expression in unique cell lines not belonging to a cVIS cannot be interpreted as the consequence of the specific retroviral integration event. Our results strongly suggest that, although the targeted genes had a crucial role in the establishment of the resistant phenotype (Van Agthoven *et al*, 1998, 2009b; Brinkman *et al*, 2000), ultimately these isogenic cell lines developed a common gene expression programme compatible with growth in the presence of tamoxifen. Additional hybridisation experiments involving transfectants with an inducible expression are required to further resolve the gene expression differences caused by the action of these genes and the underlying resistance pathways (Creighton *et al*, 2008). An alternative explanation may be that the deregulation of most targeted genes only caused post-transcriptional modifications, such as differences in the activation state of important signalling proteins, without affecting their mRNA levels. Such events cannot be measured by gene expression profiling but need to be assessed using other techniques (Korf *et al*, 2008; Schuchardt and Borlak, 2008; Speer *et al*, 2008).

The analysis of the expression data of the gene set associated with a retroviral integration within *NCOR2* in our cell model identified patients with ER*α*-positive breast cancer with different MFS (Supplementary Figure S4). The best-performing group of patients revealed by this gene signature had tumours predominantly of the luminal A, normal-like or ERBB2 molecular subtypes. These findings suggest that the genes associated with NCOR2 activation in our cell model correlate with the well-established molecular breast cancer subtypes (Perou *et al*, 2000) and thereby may provide prognostic information for ER*α*-positive breast cancer.

As viral integrations in both *NCOR2* and *CITED2* yielded differences in gene expression patterns in our cell model, we quantified their mRNA levels in breast cancer tissues to further investigate the role of these two genes in tumour aggressiveness and resistance to tamoxifen. Our results showed that in LNN patients with ER*α*-positive primary tumours who had not received systemic adjuvant treatment, high mRNA levels of *NCOR2* and *CITED2* were associated with a longer MFS independently of the traditional prognostic factors. Preliminary analyses in a subset of 120 patients for whom the molecular breast cancer subtype was known suggest that *NCOR2* and *CITED2* mRNA levels are particularly prognostic in patients with luminal A tumours, but not luminal B tumours (data not shown). In addition to its association with tumour aggressiveness, high levels of *CITED2* mRNA were also significantly associated with clinical benefit of tamoxifen treatment in advanced breast cancer independently of the traditional predictive markers including adjuvant chemotherapy. This finding further emphasises the role of *CITED2* in tamoxifen resistance.

NCOR2 participates in a co-repressor complex resulting in chromatin condensation and may also modulate ligand dependency of hormone receptors and contribute to oestrogen independency (Shou *et al*, 2004; Perissi and Rosenfeld, 2005). Furthermore, NCOR2 was shown to be a key regulator of neuronal stem cell differentiation by the repression of a specific histone demethylase (Jepsen *et al*, 2007). In a recent study by Green *et al* (2008), immunohistochemical analyses of tissue microarrays have indicated that moderate/high levels of NCOR2 protein, as found in 16.7% of tumours, are associated with poor prognosis of breast cancer patients. The differences in patient outcome, compared with the findings in our study, may be explained by the different targets measured (mRNA *vs* protein) and/or the different composition of patient groups. The study population explored by Green *et al* (2008) consisted of both ER*α*-negative and -positive tumours, patients with lymph node-positive and -negative disease, whereas patients received adjuvant systemic treatment according to their Nottingham Prognostic Index. In contrast, our study population is a homogeneous set of ER*α*-positive tumours of LNN patients who did not receive adjuvant systemic therapy, which allowed the assessment of the natural course of the disease. The importance of the selection of study groups is illustrated by the fact that we did not observe a relationship between *NCOR2* mRNA levels and MFS in patients with ER*α*-negative tumours (data not shown), contrasting with our findings in ER*α*-positive tumours (Table 2). It is noteworthy that the association of high *NCOR2* levels with a favourable outcome in breast cancer patients seems to be in conflict with our cell line data, which showed increased *NCOR2* mRNA and protein levels in tamoxifen-resistant cell lines (Figure 1 and Supplementary Figure S3). As we were unable to confirm the dominant role of NCOR2 in our cell model (Van Agthoven *et al*, 2009b) and did not establish an association with tamoxifen resistance, it is premature to speculate about mechanistic differences.

For *CITED2*, no information regarding the outcome of breast cancer patients was available. Its family member, CITED4, has been analysed in breast tumours and was found to be associated with HIF1*α* expression and to be either lost or translocated into the cytoplasm during tumour progression (Fox *et al*, 2004). CITED2 acts as a transcriptional co-factor and may regulate HIF1-stimulated apoptosis through FOXO3a (Bakker *et al*, 2007). *CITED2* expression is regulated by protein arginine methyltransferases, which modulate histone and chromatin proteins (Kleinschmidt *et al*, 2008). Loss of CITED2 has been implicated in restored sensitivity to platinum compounds in resistant ovarian cancer cells (Yanagie *et al*, 2009) and in increased invasiveness in a colon cancer model (Bai and Merchant, 2007). In line with these observations, reduced *CITED2* mRNA levels are a marker of poor prognosis in breast cancer patients. In contrast, in our cell line model, it was suggested that overexpression caused tamoxifen resistance (Figure 1). Our transfection experiments have not yet been successful in confirming this speculation (Van Agthoven *et al*, 2009b).

In summary, our functional genetic screen by retroviral insertion mutagenesis has identified several genes that are causal or strongly implicated in anti-oestrogen resistance of breast cancer cells. Although all tamoxifen-resistant cell lines obtained through this approach showed a gene expression profile distinct from parental cells, among these tamoxifen-resistant cell lines, expression patterns were nearly indistinguishable from each other. In these cases, alteration of signalling cascades leading to tamoxifen resistance may occur at the post-transcriptional level, such as protein activation status, which is not translated into different gene expression patterns. In contrast, cell lines with viral integrations affecting *NCOR2* and *CITED2* expression, showed different gene expression patterns when compared with the other tamoxifen-resistant cell lines. Importantly, our experiments have shown that *NCOR2, CITED2* and other genes previously revealed through functional screening (Van Agthoven *et al*, 2009a) are implicated in breast cancer outcome and therefore bear clinical relevance. On the basis of these results, further studies into the mechanisms of tamoxifen resistance of these cell line models are warranted to identify novel targets for therapy, as well as novel prognostic and predictive markers. Ultimately, this could result in improved outcome to endocrine therapy and to a more individualised management of breast cancer patients.

## Figures and Tables

**Table 1:** Progression-free survival (PFS) and clinical benefit after first-line tamoxifen treatment of 296 patients with ER+ primary breast tumours

		**PFS^a^**						**Clinical benefit**					
		**Univariate**			**Multivariate**			**Univariate**			**Multivariate**		
	***N***	**HR**	**95% CI**	***P*-value**	**HR**	**95% CI**	***P*-value**	**OR**	**95% CI**	***P*-value**	**OR**	**95% CI**	***P*-value**
*Age at start of therapy (years)*				0.048			NS			NS			NS
⩽40	17	1			1			1			1		
41–55	100	0.69	0.37–1.30		0.62	0.32–1.19		0.89	0.31–2.53		0.81	0.26–2.52	
56–70	105	0.59	0.31–1.11		0.54	0.24–1.25		1.34	0.47–3.82		0.88	0.23–3.40	
>70	74	0.41	0.21–0.82		0.38	0.16–0.92		1.46	0.49–4.30		0.99	0.25–3.98	
													
*Menopausal status at start of therapy*				NS			NS			0.096			NS
Pre	77	1			1			1			1		
Post	219	0.74	0.52–1.06		1.07	0.63–1.81		1.57	0.92–2.66		1.27	0.55–2.94	
													
*Disease-free interval (years)*				<0.001			<0.001			<0.001			<0.001
⩽1	74	1			1			1			1		
1–3	134	0.46	0.32–0.67		0.48	0.33–0.71		3.52	1.94–6.38		3.49	1.88–6.46	
>3	88	0.39	0.25–0.61		0.40	0.26–0.64		3.91	2.03–7.55		3.89	1.95–7.77	
													
*Dominant site of relapse*				NS			NS			NS			NS
L R	33	1			1			1			1		
Bone	154	1.24	0.70–2.19		1.21	0.66–2.20		0.63	0.28–1.41		0.57	0.24–1.38	
Viscera	109	1.17	0.65–2.11		1.28	0.69–2.37		0.81	0.35–1.88		0.65	0.26–1.62	
													
*ESR1 mRNA level*													
Continuous		0.87	0.80–0.94	<0.001	0.88	0.81–0.96	0.005	1.21	1.08–1.36	0.001	1.20	1.05–1.37	0.007
													
*PGR mRNA level*													
Continuous		0.93	0.86–1.01	0.087	0.95	0.87–1.03	0.021	1.11	0.99–1.25	0.079	1.06	0.93–1.21	NS
													
**Factors analysed**					**Additions to the base model^b^**						**Additions to the base model^b^**		
*NCOR2*													
Continuous		1.06	0.84–1.34	NS	1.12	0.89–1.41	NS	0.88	0.63–1.23	NS	0.79	0.55–1.13	NS
Median		1.13	0.81–1.57	NS	1.19	0.85–1.65	NS	0.87	0.54–1.39	NS	0.77	0.46–1.28	NS
													
*CITED2*													
Continuous		0.84	0.70–1.00	0.058	0.94	0.77–1.14	NS	1.34	1.03–1.76	0.028	1.17	0.86–1.59	NS
Median		0.58	0.42–0.81	0.001	0.65	0.46–0.93	0.017	2.20	1.36–3.57	0.001	1.91	1.12–3.25	0.017

Abbreviations: CI=confidence interval; HR=hazard ratio; NS=*P*-values >0.10; L R=local regional; OR=odds ratio.

a PFS was censored at 9 months, to avoid violation of the proportional hazards assumption.

b Factors were separately introduced to the base multivariate model that included the factors age, menopausal status, disease-free interval, dominant site of relapse, *ESR1* and *PGR* mRNA levels.

**Table 2:** Metastasis-free survival (MFS) and overall survival (OS) of 620 lymph node-negative patients with ER+ primary breast tumours

	**MFS**						**OS**					
	**Univariate**			**Multivariate**			**Univariate**			**Multivariate**		
	**HR**	**95% CI**	***P*-value**	**HR**	**95% CI**	***P*-value**	**HR**	**95% CI**	***P*-value**	**HR**	**95% CI**	***P*-value**
*Age (years)*			0.005			0.066			NS			NS
⩽40	1			1			1			1		
41–55	0.76	0.50–1.14		0.81	0.53–1.24		0.88	0.55–1.41		0.92	0.57–1.50	
56–70	0.54	0.35–0.83		0.45	0.24–0.87		0.80	0.49–1.30		0.61	0.3–1.26	
>70	0.48	0.29–0.78		0.39	0.19–0.78		1.20	0.73–1.97		0.95	0.45–2.00	
												
*Menopausal status*			0.013			NS			NS		NS	
Pre	1			1			1			1		
Post	0.71	0.54–0.93		1.08	0.64–1.81		1.12	0.84–1.50		1.24	0.7–2.20	
												
*Tumour size*			0.074			NS			NS			NS
⩽2 cm	1			1			1			1		
>2 cm	1.28	0.98–1.67		1.26	0.95–1.66		1.24	0.93–1.64		1.14	0.85–1.53	
												
*Grade*			0.002			0.002			0.069			NS
Poor	1			1			1			1		
Unknown	1.03	0.77–1.38		1.13	0.83–1.52		1.01	0.74–1.39		1.03	0.74–1.42	
Moderate/good	0.53	0.35–0.79		0.55	0.36–0.83		0.65	0.44–0.98		0.70	0.47–1.06	
												
*ESR1 mRNA level*												
Continuous	0.96	0.91–1.02	NS	1.06	0.99–1.13	NS	0.99	0.93–1.05	NS	1.03	0.96–1.11	NS
*PGR* mRNA level												
Continuous	0.91	0.85–0.97	0.003	0.90	0.84–0.96	0.002	0.89	0.83–0.95	0.001	0.88	0.81–0.94	<0.001
												
**Factors analysed**				**Additions to the base model^a^**						**Additions to the base model^a^**		
*NCOR2*												
Continuous	0.63	0.50–0.78	<0.001	0.67	0.54–0.83	<0.001	0.75	0.60–0.95	0.016	0.78	0.62–0.98	0.032
Median	0.62	0.47–0.82	0.001	0.68	0.51–0.89	0.006	0.73	0.55–0.97		0.031	0.78–0.58	1.04
*CITED2*												
Continuous	0.69	0.58–0.84	<0.001	0.77	0.63–0.93	0.008	0.90	0.74–1.09	NS	0.90	0.73–1.11	NS
Median	0.62	0.47–0.82	0.001	0.71	0.53–0.94	0.017	0.73	0.55–0.97	0.028	0.75	0.55–1.01	0.054

Abbreviations: CI=confidence interval; HR=hazard ratio; NS=*P*-value >0.10.

a Factors were separately introduced to the base multivariate model that included the factors age, menopausal status, tumour size, grade, *ESR1* and *PGR* mRNA levels.
