# Supplementary material for: CITED2 and NCOR2 in anti-oestrogen resistance and progression of breast cancer
Source: Br J Cancer. 2009 Nov 10;101(11):1824–32. doi: 10.1038/sj.bjc.6605423 (PMC2788259; doi:10.1038/sj.bjc.6605423)
Supplement: Supplementary information [file 6605423x1.pdf]

Supplementary Information

Materials and Methods

Preparation of RNA and hybridization probes.

Total RNA was prepared from approximately 80% confluent cells grown in 25 or 75 cm^2 tissue culture flasks (Corning Inc., Corning, NY) by direct lysis with RNA-Bee™ (Bio-connect, Huissen, NL) according to the protocol of the supplier. RNA quality was checked by agarose gel electrophoresis. cDNA synthesis was performed using an T7dT-oligo primer (5' GGCCAGTGAATTG TAATACGACTCACTATAGGGAGGCGG (T24) 3' (Baugh *et al*, 2001), 3 μg of total RNA and reverse transcriptase (Superscript II, Invitrogen, Breda, The Netherlands). Reaction was performed in the presence of trehalose (Fluka, Sigma-Aldrich), RNAsin or Rnase OUT (Invitrogen) and deoxy-nucleotides. The second strand synthesis was performed using E.coli ligase, E.coli DNA polymerase (Invitrogen) and RNase H (Promega Benelux b.v., Leiden, The Netherlands). The double stranded cDNA was purified on Quiaquick PCR columns (Qiagen, Hilden, Germany). In vitro transcription using the T7 Megascript Kit (Ambion, Austen, Texas, USA) was used to produce amplified RNA (aRNA), which was purified using Rneasy columns (Qiagen). As a reference, aRNA was prepared of total RNA from a mixture of cell lines as described previously (Jansen *et al*, 2005).

Gene expression profiling

Spotted oligo microarrays with the Operon V3.0 library (35K Human, http://omad.operon.com/humanV3) were obtained from the Netherlands Cancer Institute Central Microarray Facility (NKI-CMF). Protocols for sample preparation were taken from the NKI-CMF website (http://microarrays.nki.nl) and detailed elsewhere (Meester-Smoor *et al*, 2008). In short, 1 μg of amplified RNA was labelled using the ULS™-Cy3/5 aRNA fluorescent labelling kit (Kreatech, Amsterdam, The Netherlands), and was used for hybridization on the same day. The labelling efficiency was checked on a NanoDrop instrument (Isogen Life Sciences B.V. De Meern, The Netherlands). The labelled aRNA was fragmented (RNA Fragmentation Reagents, Ambion) and mixed with blocking solution containing Poly d(A), Cot-1 DNA, and yeast t-RNA (GE Healthcare, Zeist, the Netherlands and Roche, Basel, Switzerland). The arrays were hybridized overnight at 42°C on a Tecan HS4800 hybridization station, according to the M016 protocol developed by the Erasmus Center for Biomics (http://www.biomics.nl). Two independent samples of a cell line were hybridized against the reference and one sample was also used in a dye-swap hybridization. The hybridized arrays were scanned on a ScanArray Express HT instrument (Perkin Elmer Life and Analytical Sciences BV, Groningen, The Netherlands). The measured fluorescence intensities were determined using ImaGene software version 6.0 (Biodiscovery, El Segundo, USA). The ImaGene data were uploaded into the CMF database (CMFdb, http://cmfdb.nki.nl) and normalized using the lowess subarray method (default settings). The normalized data were downloaded from the CMF database and further analyzed. Both raw and normalized data have been deposited in NCBI's Gene Expression Omnibus (Edgar *et al*, 2002 and are accessible through GEO Series accession number GSE14513 (http://www.ncbi.nlm.nih.gov/geo/query/acc.cgi?acc=GSE14513).

Supplementary Information

Bioinformatic analysis

Analyses on normalized data were performed using BRB-ArrayTools developed by Dr. Richard Simon and BRB-ArrayTools Development Team (http://linus.nci.nih.gov/BRB-ArrayTools.html). Cell line hybridization data were used individually without averaging over the replicates and dye swap. Spots were excluded when significantly associated with dye-swapping (57%), or more than 80% missing data (13%). Unsupervised hierarchical clustering was performed using 15376 spots meeting the selection criteria mentioned above. Class comparisons (significance threshold in univariate tests <0.001) were performed with the cell lines grouped according to their common virus integration site (cVIS) (Van Agthoven *et al*, 2009) using all spots. The cell lines with a viral integration in *BCAR1* or *BCAR3* were used as the reference group for the class comparison analyses with each of the other groups of cell lines. A set of 1106 spots (approximately 750 unique annotated genes, Table S1) was obtained which reflect the major differences between all classes of cell lines. Spots strongly associated with the dye-swap were eliminated from this list. Hierarchical clustering was performed with 251 selected spots using Spotfire DecisionSite 9.0 (Tibco, Somerville, USA). Genes (n=265) significantly associated with an integration within *NCOR2* (class comparison analysis, see above) were linked to Affymetrix probe-sets using Entrez Gene and Ensemble identifiers (Table S2) to facilitate their evaluation in human breast cancer micro-array data (Wang *et al*, 2005).

References

Baugh LR, Hill AA, Brown EL, Hunter CP. (2001). Quantitative analysis of mRNA amplification by in vitro transcription. *Nucleic Acids Res*, **29,** E29.

Edgar R, Domrachev M, Lash AE. (2002). Gene Expression Omnibus: NCBI gene expression and hybridization array data repository. *Nucleic Acids Res*, **30,** 207-210.

Jansen MP, Foekens JA, van Staveren IL, Dirkzwager-Kiel MM, Ritstier K, Look MP, Meijer-van Gelder ME, Sieuwerts AM, Portengen H, Dorssers LC, Klijn JG, Berns EM. (2005). Molecular classification of tamoxifen-resistant breast carcinomas by gene expression profiling. *J Clin Oncol*, **23,** 732-740.

Meester-Smoor MA, Janssen MJ, Grosveld GC, de Klein A, van IWF, Douben H, Zwarthoff EC. (2008). MN1 affects expression of genes involved in hematopoiesis and can enhance as well as inhibit RAR/RXR-induced gene expression. *Carcinogenesis*, **29,** 2025-2034.

Perou CM, Sorlie T, Eisen MB, van de Rijn M, Jeffrey SS, Rees CA, Pollack JR, Ross DT, Johnsen H, Akslen LA, Fluge O, Pergamenschikov A, Williams C, Zhu SX, Lonning PE, Borresen-Dale AL, Brown PO, Botstein D. (2000). Molecular portraits of human breast tumours. *Nature*, **406,** 747-752.

Sotiriou C, Wirapati P, Loi S, Harris A, Fox S, Smeds J, Nordgren H, Farmer P, Praz V, Haibe-Kains B, Desmedt C, Larsimont D, Cardoso F, Peterse H, Nuyten D, Buyse M, Van de Vijver MJ, Bergh J, Piccart M, Delorenzi M. (2006). Gene expression profiling in breast cancer: understanding the molecular basis of histologic grade to improve prognosis. *J Natl Cancer Inst*, **98,** 262-272.

Wang Y, Klijn JG, Zhang Y, Sieuwerts AM, Look MP, Yang F, Talantov D, Timmermans M, Meijer-van Gelder ME, Yu J, Jatkoe T, Berns EM, Atkins D, Foekens JA. (2005). Gene-expression profiles to predict distant metastasis of lymph-node-negative primary breast cancer. *Lancet*, **365,** 671-679.

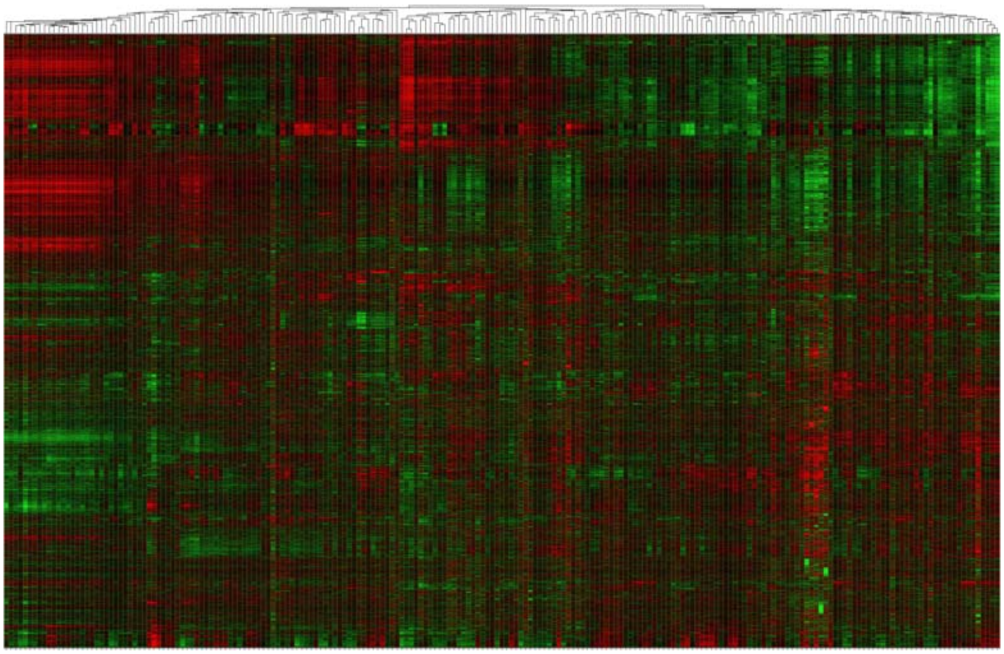

Figure S1 Hierarchical clustering of 15376 genes in 69 tamoxifen-resistant breast cancer cell lines
Clustering was performed using the unweighted pair-group method with arithmetic mean in Spotfire. Data of 219 hybridization experiments are presented in the columns. In the rows, expression of 15376 genes above the mean is shown in red, below de mean is indicated in green.

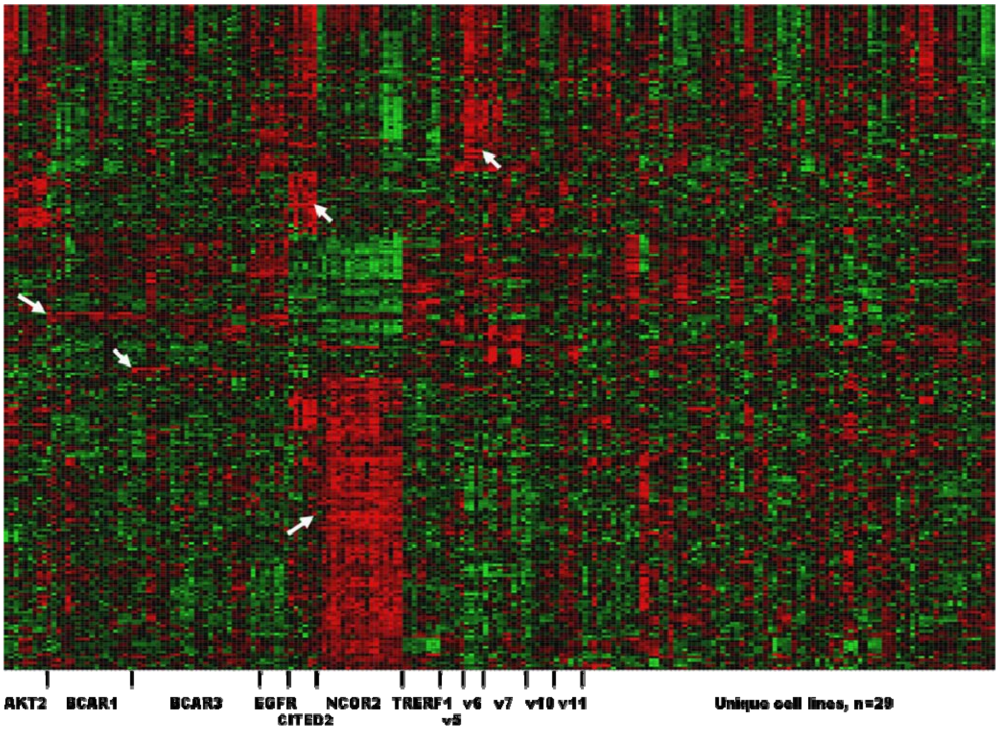

Figure S2 Hierarchical clustering of 251 genes in tamoxifen-resistant breast cancer cell lines
All cell lines were grouped according to the location of the retrovirus in the cellular genome (columns). cVIS has been abbreviated to v. Cell lines not belonging to a cVIS are grouped under “Unique cell lines”. Gene clustering was performed using the unweighted pair-group method with arithmetic mean in Spotfire. Expression of 251 selected genes above the mean is shown in red, below the mean is indicated in green. Target genes in the integration loci are marked with arrows (from left to right: *BCAR1*, *BCAR3*, *CITED2*, *NCOR2*, and *TSHZ1*). Gene identifiers and the order in the heat map are provided in Table S1.

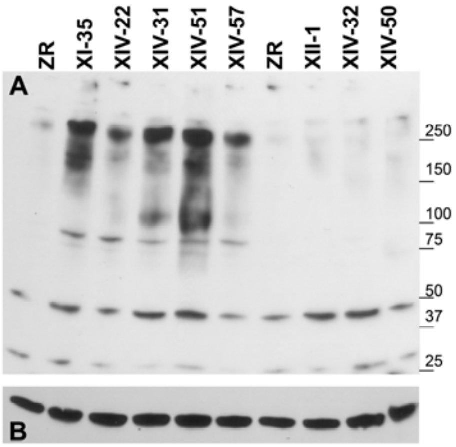

Figure S3 Western blot analysis of cell lines
Total lysates of cell lines with an integration in the *NCOR2* locus (XI-35, XIV-22, XIV-31, XIV-51 and XIV-57), cell lines with an integration elsewhere, and the parental ZR-75-1 cells (ZR) were blotted and probed with antibodies directed against NCOR2 (ab24551, Abcam, panel A) or against β-actin (Sigma, panel B). Positions of marker proteins (in kDa) are given on the right.

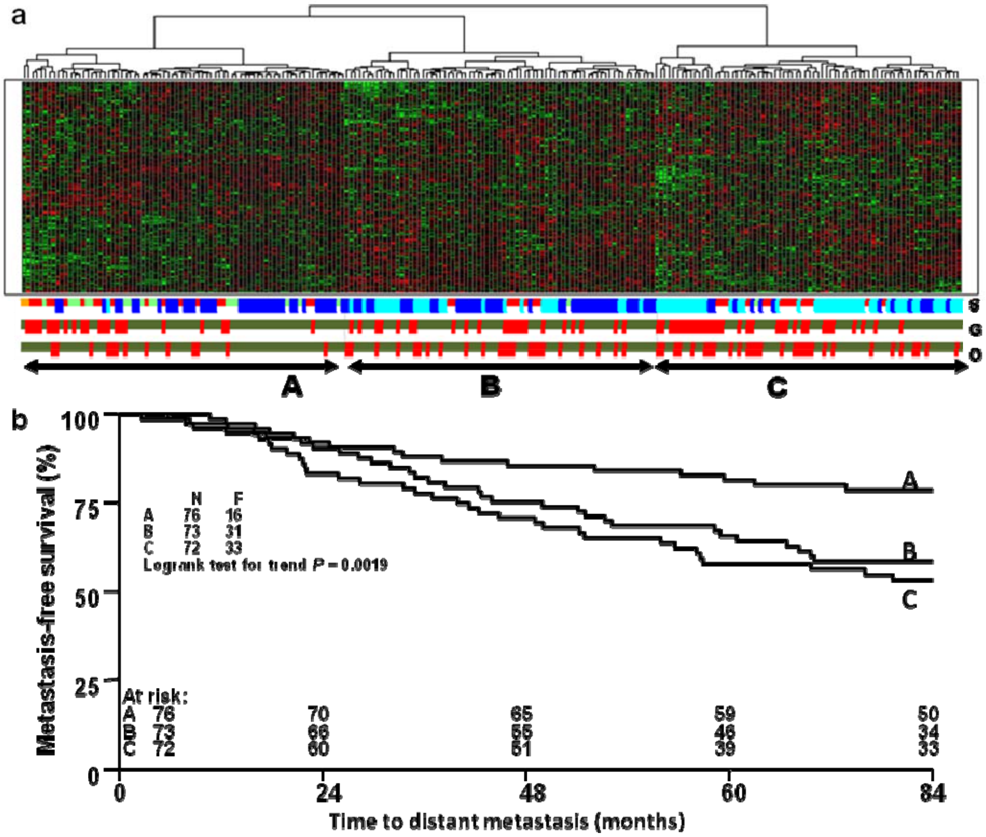

Figure S4 Relevance of NCOR2 gene signature in 221 ER-positive breast cancers of lymph node-negative patients
a. Expression data of 171 unique genes corresponding to the gene set differentially expressed in cell lines with a retroviral integration in *NCOR2* were recovered from 221 *ESR1* mRNA-positive specimens of LNN breast cancer patients who had not received systemic adjuvant treatment (Wang et al, 2005). Clustering was performed using Ward's method in Spotfire. Affymetrix probe-sets ID are given in Supplementary Table S2. Further information is indicated by coloured bars: molecular subtype according to Perou et al, 2000) (S: dark blue = luminal A, light blue = luminal B, red = ERBB2, orange = basal, green = normal like), molecular grade according to Sotiriou et al, 2006) (G: red = poor, green = good) and outcome (O: red = <5 years, green = >5 years). Tumours were divided into three groups (A, B and C) according to the cluster dendrogram as indicated.
b. Kaplan-Meier curves for MFS for the three subgroups of patients based on the classification of the samples according to the hierarchical gene clustering (a). Patients at risk at 24-month intervals are indicated. N, number of patients; F, number of recurrences.

Table S1. Spotted oligonucleotides selected by class comparison and used for clustering

All spots selected using class comparison analysis with BCAR1/BCAR3 cell lines as reference group

ID	ID GPL4035
251 list	Spots left after visual inspection and used for hierarchical clustering of the cell lines (numbers reflect hierarchical cluster order in Figures 1 and S2)
Gene	Gene identifier, Entrez gene, Ensembl gene or h-invitational cluster (http://microarrays.nki.nl/services/OperonHuman.html)
	No more than 2 gene identifiers or gene symbols are listed

ID	251 list	Gene	Gene Symbol
32372	1	N/A	
15365	2	ENSG00000071082	RPL31P10
36569	3	ENSG00000213302	
2383	4	ENSG00000179131	
19100	5	ENSG00000213942	
19835	6	100130130	
6528	7	ENSG00000213657	
7061	8	ENSG00000213598	
9018	9	ENSG00000213849	
8429	10	ENSG00000213470	ATF7
17348	11	391126	
152	12	ENSG00000161970	RPL26
24847	13	730070	
9493	14	ENSG00000213098	
26593	15	ENSG00000175333	dJ612B15.1
29555	16	ENSG00000143947	RPS27A
29290	17	ENSG00000143947	
11633	18	ENSG00000213432	RPL17 dJ612B15.1
933	19	ENSG00000214878	
14917	20	ENSG00000122406	RPL5
27148	21	ENSG00000140740	UQCRC2
1085	22	ENSG00000184319	MGC70863
23053	23	ENSG00000213753	
34305	24	ENSG00000174748	RPL15
18083	25	ENSG00000115685	PPP1R7
5906	26	N/A	
22224	27	ENSG00000127720	C12orf26
30262	28	ENSG00000119705	C14orf156
29133	29	ENSG00000156928	C7orf30
15467	30	ENSG00000145425	RPS3A
27302	31	N/A	
34029	32	ENSG00000183920	EEF1A1
28732	33	ENSG00000177173	
10528	34	ENSG00000027001	MIPEP
14530	35	ENSG00000089818	NECAP1
18906	36	ENSG00000174486	
23911	37	ENSG00000074201	CLNS1A
26676	38	ENSG00000130177	CDC16
29583	39	ENSG00000143870	PDIA6
24089	40	ENSG00000198875	
14694	41	ENSG00000118363	SPCS2
4933	42	ENSG00000120963	ZNF706
35810	43	ENSG00000088325	TPX2
24097	44	ENSG00000136938	ANP32B
28545	45	ENSG00000155561	NUP205
15366	46	ENSG00000118816	CCNI
36715	47	ENSG00000197024	ZNF398
34069	48	ENSG00000090889	KIF4A
28099	49	ENSG00000143653	SCCPDH
6464	50	ENSG00000110092	CCND1
3323	51	ENSG00000125844	RRBP1
9621	52	ENSG00000137804	NUSAP1
21128	53	ENSG00000139291	TMEM19
23626	54	ENSG00000121152	NCAPH
18769	55	ENSG00000166483	WEE1
18342	56	ENSG00000179981	TSHZ1
32092	57	N/A	
15294	58	ENSG00000212653	C6orf216 GUSB
37475	59	ENSG00000162545	CAMK2N1
25072	60	ENSG00000181571	
35901	61	ENSG00000197391	
35849	62	N/A	PTMA
10532	63	ENSG00000187514	PTMA
23049	64	ENSG00000197272	IL27
34447	65	ENSG00000212919	PIGC
16006	66	ENSG00000134905	FLJ12118
28688	67	ENSG00000147117	ZNF157
7208	68	ENSG00000160767	C1orf2
9728	69	ENSG00000160179	ABCG1
35935	70	ENSG00000162520	SYNC1
31717	71	ENSG00000095739	BAMBI
32218	72	ENSG00000135346	CGA
22080	73	ENSG00000141668	CBLN2
25849	74	ENSG00000204776	
1463	75	ENSG00000183038	
37474	76	ENSG00000164442	CITED2
35934	77	ENSG00000164411	GJB7
26774	78	ENSG00000174446	SNAPC5
12436	79	ENSG00000213924	
30513	80	ENSG00000135477	
32039	81	ENSG00000170430	MGMT
20811	82	ENSG00000171246	NPTX1
14815	83	54815	
7460	84	ENSG00000188783	PRELP
15786	85	ENSG00000121270	ABCC11
32524	86	ENSG00000147853	AK3
33472	87	ENSG00000048707	VPS13D
25797	88	ENSG00000166920	C15orf48
12102	89	ENSG00000173467	BCMP11
28403	90	ENSG00000163993	S100P
20942	91	ENSG00000086548	CEACAM6
31239	92	ENSG00000086548	CEACAM6
462	93	ENSG00000163435	ELF3
6734	94	ENSG00000163435	ELF3
5308	95	ENSG00000140391	TSPAN3
29421	96	ENSG00000082146	ALS2CR2
28924	97	ENSG00000005893	LAMP2
23503	98	ENSG00000162496	DHRS3
18107	99	ENSG00000131981	LGALS3
30651	100	ENSG00000131981	LGALS3
1754	101	ENSG00000135052	C9orf155
26591	102	ENSG00000130513	GDF15
33139	103	ENSG00000146242	TPBG
34114	104	ENSG00000164904	ALDH7A1
12263	105	ENSG00000067182	TNFRSF1A
1873	106	ENSG00000102243	VGLL1
28280	107	ENSG00000051128	HOMER3
10139	108	ENSG00000213406	ANXA2P1
14388	109	ENSG00000213977	P2RX5
91	110	ENSG00000187653	
28789	111	ENSG00000101997	CACNA1F
20158	112	ENSG00000081870	C1orf41
14346	113	ENSG00000166289	PLEKHF1
21812	114	ENSG00000002834	LASP1
3378	115	ENSG00000137767	SQRDL
36277	116	ENSG00000137767	SQRDL
34664	117	ENSG00000050820	BCAR1
8149	118	ENSG00000181649	PHLDA2
20665	119	ENSG00000181649	PHLDA2
35575	120	ENSG00000125144	MT1F
9836	121	ENSG00000168306	ACOX2
7553	122	ENSG00000213866	YBX1
16139	123	4904	
26490	124	ENSG00000213866	YBX1
6855	125	ENSG00000113269	RNF130
28084	126	ENSG00000002834	LASP1
8816	127	ENSG00000108679	LGALS3BP
27292	128	ENSG00000124935	SCGB1D2
18840	129	ENSG00000110484	SCGB2A2
27958	130	ENSG00000148175	STOM
28207	131	ENSG00000162174	ASRGL1
16141	132	ENSG00000205592	MUC19
34174	133	ENSG00000182795	C1orf116
22777	134	ENSG00000156853	ZNF689
33143	135	ENSG00000124713	GNMT
27902	136	ENSG00000182795	C1orf116
10236	137	ENSG00000198799	LRIG2
17517	138	ENSG00000137936	BCAR3
4713	139	ENSG00000186187	ZNRF1
30707	140	ENSG00000187446	CHP
20023	141	ENSG00000187446	CHP
29924	142	ENSG00000129757	CDKN1C
32579	143	ENSG00000144857	BOC
5055	144	ENSG00000145632	PLK2
25545	145	N/A	
13738	146	ENSG00000134107	BHLHB2
9840	147	ENSG00000160161	CILP2
1870	148	ENSG00000120129	DUSP1
21195	149	ENSG00000077327	SPAG6
14923	150	ENSG00000077327	SPAG6
22837	151	ENSG00000138650	PCDH10

Table S1 continued

ID	251 list	Gene	Gene Symbol
31841	152	ENSG00000198535	NLF1
23323	153	ENSG00000067798	NAV3
17051	154	ENSG00000067798	NAV3
18830	155	ENSG00000153002	CPB1
18469	156	ENSG00000136770	DNAJC1
4101	157	ENSG00000155368	DBI
35629	158	ENSG00000155368	DBI
34771	159	ENSG00000196428	TSC22D2
5587	160	23089	PEG10
18723	161	10439	OLFM1
32181	162	ENSG00000134250	NOTCH2 NOTCH2NL
22496	163	ENSG00000215845	F11R
28177	164	ENSG00000166347	CYB5A
6208	165	ENSG00000124399	
5441	166	ENSG00000133193	COG1
16968	167	ENSG00000010278	CD9
36014	168	ENSG00000172889	EGFL7
33226	169	ENSG00000137100	DCTN3
36908	170	ENSG00000196998	WDR45
9685	171	ENSG00000196998	WDR45
33782	172	ENSG00000187134	AKR1C1
15956	173	N/A	
14311	174	ENSG00000151632	AKR1C2
4826	175	ENSG00000166033	HTRA1
31460	176	ENSG00000162685	
31411	177	ENSG00000116670	MAD2L2
2459	178	ENSG00000115266	APC2
22243	179	ENSG00000205544	C17orf61
17709	180	ENSG00000183648	NDUFB1
1436	181	ENSG00000167123	CEECAM1
22958	182	ENSG00000092445	TYRO3
37144	183	ENSG00000149809	TM7SF2
25172	184	ENSG00000054148	PHPT1
36817	185	ENSG00000100097	LGALS1
5345	186	ENSG00000100097	LGALS1
26715	187	ENSG00000177239	
18740	188	ENSG00000167468	GPX4
31284	189	ENSG00000167468	GPX4
30137	190	ENSG00000179271	GADD45GIP1
37371	191	ENSG00000130725	UBE2M
29721	192	ENSG00000152082	FAM128B
13726	193	ENSG00000173272	
15388	194	ENSG00000196498	NCOR2
10142	195	ENSG00000115274	WBP1
28248	196	ENSG00000110011	DNAJC4
8499	197	ENSG00000144579	CTDSP1
9639	198	ENSG00000213603	STMN3
34839	199	ENSG00000213603	STMN3
25293	200	ENSG00000181222	POLR2A
22024	201	ENSG00000143258	USP21
31833	202	ENSG00000198276	UCKL1
11725	203	ENSG00000123144	C19orf43
20619	204	442421	
30036	205	ENSG00000127838	PNKD
7973	206	ENSG00000110011	DNAJC4
20681	207	ENSG00000143355	LHX9
35548	208	ENSG00000141314	RHBDL3
12449	209	ENSG00000135638	EMX1
11432	210	ENSG00000168243	GNG4
4441	211	ENSG00000136630	HLX1
29620	212	ENSG00000104979	C19orf53
9399	213	ENSG00000130288	NDUFA13
33423	214	ENSG00000204316	MRPL38
10102	215	ENSG00000118050	C19orf24
36341	216	ENSG00000102030	ARD1A
23414	217	ENSG00000172922	RNASEH2C
5343	218	ENSG00000111678	C12orf57
27427	219	ENSG00000143409	FAM63A
37629	220	ENSG00000099875	MKNK2
8892	221	ENSG00000179044	NOL3
30629	222	ENSG00000103227	TMEM112
6448	223	ENSG00000169738	DCXR
15143	224	ENSG00000113763	UNC5A
6336	225	ENSG00000169750	RAC3
1372	226	ENSG00000008382	FLJ14981
27832	227	ENSG00000019169	MARCO
29572	228	ENSG00000184451	CCR10
2126	229	ENSG00000086504	MRPL28
20041	230	ENSG00000076604	TRAF4
3077	231	ENSG00000099377	HSD3B7
4698	232	ENSG00000117318	ID3
17772	233	ENSG00000125968	ID1
36629	234	146691	TOM1L2
32116	235	ENSG00000007541	PIGQ
11245	236	ENSG00000137880	GCHFR
4690	237	ENSG00000148362	C9orf142
33711	238	ENSG00000064961	HMG20B
24877	239	348262	LOC348262
1241	240	ENSG00000197766	CFD
13902	241	N/A	
36986	242	ENSG00000157306	
18022	243	ENSG00000185956	
15949	244	ENSG00000137959	IFI44L
17520	245	ENSG00000127603	MACF1
30575	246	ENSG00000063241	ISOC2
36840	247	8364	HIST1H4C
31987	248	ENSG00000215809	EDARADD
16957	249	ENSG00000136718	IMP4
13625	250	ENSG00000142208	AKT1
21092	251	ENSG00000167767	KRT80
1		N/A	
4		595135, 727880	
25		ENSG00000104408	EIF3S6
107		N/A	QSCN6
119		ENSG00000180922	
144		ENSG00000177971	IMP3
164		ENSG00000110321	EIF4G2
169		ENSG00000181991	MRPS11
170		N/A	
226		ENSG00000213007	
268		ENSG00000141934	PPAP2C
291		727880, 729348	
340		ENSG00000211451	
354		ENSG00000136026	CKAP4
380		ENSG00000149100	PCID1
396		ENSG00000166783	KIAA0430
398		ENSG00000215042	
538		100129791	BNIP3L ARHGAP15
667		ENSG00000111907	TPD52L1
761		ENSG00000214160	ALG3
925		ENSG00000101752	MIB1
954		ENSG00000206413	
976		ENSG00000108771	LGP2
1099		ENSG00000171490	RSL1D1
1109		N/A	
1138		ENSG00000122597	
1144		ENSG00000107164	FUBP3
1171		N/A	CSNK1A1
1195		ENSG00000131558	EXOC4
1346		ENSG00000196542	C3orf57
1363		ENSG00000112081	SFRS3
1474		ENSG00000185163	
1542		ENSG00000187504	
1546		ENSG00000180922	
1646		ENSG00000121774	KHDRBS1
1896		ENSG00000128510	CPA4
1937		ENSG00000069431	
2070			
2080		113157	RPLP0P2
2123		ENSG00000113318	MSH3
2125		ENSG00000101158	TH1L
2130		N/A	
2203		ENSG00000131143	COX4I1
2272		ENSG00000009413	
2287		ENSG00000131236	CAP1
2323		ENSG00000065154	OAT
2326		100131707, 729348	ARHGAP15
2330		729348, 100129791	BNIP3L
2433		606724	LOC606724
2439		ENSG00000179085	DPM3
2487		ENSG00000115459	RBED1
2491		ENSG00000077254	USP33
2680		ENSG00000196976	LAGE3
2691		ENSG00000215839	
2966			
2972		ENSG00000180922	
3048		ENSG00000104447	TRPS1
3068		ENSG00000138434	SSFA2
3102		ENSG00000116906	GNPAT
3152		ENSG00000143162	CREG1
3228		ENSG00000100121	
3338		ENSG00000160844	
3475		ENSG00000196720	
3496		ENSG00000108061	SHOC2
3562		ENSG00000173726	TOMM20
3573		ENSG00000213007	
3582		ENSG00000089505	CKLF
3584		ENSG00000012779	ALOX5
3605		ENSG00000213585	VDAC1
3611		ENSG00000069275	NUCKS1

Table S1 continued

**ID**	**251 list**	**Gene**	**Gene Symbol**
3661		ENSG00000062716	TMEM49
3679		727880	
3698		ENSG00000183888	C1orf64
3705		ENSG00000213061	
3713		ENSG00000151012	
3740		ENSG00000140416	TPM1
3820		ENSG00000158747	NBL1
3831		ENSG00000111596	CNOT2
3880		ENSG00000100028	SNRPD3
3881		ENSG00000135821	GLUL
3899		ENSG00000170266	GLB1
3998		ENSG00000120885	CLU
4035		ENSG00000180922	
4051		ENSG00000129007	
4112		ENSG00000109113	RAB34
4132		ENSG00000153443	FAM100A
4138		ENSG00000120853	GOLGA2L1
4341		ENSG00000073905	
4354		ENSG00000180922	MIPOL1 FLJ20298
4420		ENSG00000105926	MPP6
4443		ENSG00000130748	TMEM160
4542		ENSG00000213365	LOC401703
4559		ENSG00000111321	LTBR
4672		N/A	
4717		ENSG00000157214	STEAP2
4731		ENSG00000079459	FDFT1
4777		ENSG00000138821	SLC39A8
4821		N/A	
4890		ENSG00000134759	STATIP1
4914		ENSG00000150656	CNDP1
4935		ENSG00000171763	SPATA5L1
5129		ENSG00000079432	CIC
5185		ENSG00000204794	
5209		ENSG00000212852	HS6ST3
5409		ENSG00000196977	
5420		ENSG00000139055	C12orf46
5565		ENSG00000196683	TOMM7
5685		N/A	
5718		ENSG00000189186	
5839		ENSG00000213007	
5854			CANX
5866			CANX
5899		ENSG00000139579	
5926		645694, 727880	
5932		ENSG00000105983	LMBR1
5996		ENSG00000069431	
6190			CANX
6193		595135, 727880	BNIP3L ARHGAP15
6222		ENSG00000129680	
6345		ENSG00000139318	DUSP6
6348		N/A	
6390		ENSG00000174453	
6391		ENSG00000183305	MAGEA2B
6452		ENSG00000198003	MGC20983
6480		ENSG00000204600	FLJ45422
6621		ENSG00000213007	ADAMTSL3
6667		ENSG00000211451	LOC144766
6688		ENSG00000106772	KIAA0367
6745		N/A	
6769		ENSG00000070190	
6772		ENSG00000196433	ASMT
6920		ENSG00000124496	
6927		440737	
6971		ENSG00000064763	MLSTD1
7019		ENSG00000131171	SH3BGRL
7039		ENSG00000187554	TLR5
7168		ENSG00000041353	RAB27B
7194		ENSG00000090975	PITPNM2
7247		ENSG00000115350	POLE4
7258		729348	BNIP3L
7287		ENSG00000171475	WIPF2
7328		ENSG00000126790	C14orf149
7384		ENSG00000198681	MAGEA1
7396		ENSG00000214366	
7443		ENSG00000113712	CSNK1A1
7635		ENSG00000206232	PSMB9
7883		ENSG00000147604	
7997		ENSG00000138095	LRPPRC
8011		ENSG00000069431	LOC285690 DSCR3
8025		ENSG00000138069	RAB1A
8027		ENSG00000126005	ITGB4BP
8357		ENSG00000154328	NEIL2
8391		ENSG00000114902	SPCS1
8406		N/A	TPTE2
8418		ENSG00000148331	
8435		ENSG00000170540	ARL6IP1
8462		ENSG00000173153	ESRRA
8475		ENSG00000131148	COX4NB
8582		ENSG00000149260	CAPN5
8684		ENSG00000177665	
8740		ENSG00000198397	
8758		ENSG00000120265	PCMT1
8841		ENSG00000104419	NDRG1
8842		ENSG00000120438	TCP1
8952		ENSG00000126903	SLC10A3
8966		729348	ARHGAP15
8997		ENSG00000178718	RPP25
9002			RPLP1
9031		ENSG00000214289	AMOTL2
9156		ENSG00000009335	UBE3C
9193		ENSG00000196333	LOC285690
9291		ENSG00000110717	NDUFS8
9315		ENSG00000130227	XPO7
9320		ENSG00000104447	TRPS1
9366		ENSG00000136682	CBWD1 LOC220869
9437		N/A	
9445		ENSG00000166794	PPIB
9465		ENSG00000181904	C5orf24
9489		ENSG00000185164	NOMO1 NOMO2
9564		ENSG00000141582	CBX4
9622		ENSG00000134716	CYP2J2
9643		ENSG00000203365	FLJ44796
9720		N/A	
9745		ENSG00000180922	
9764		ENSG00000127870	RNF6
9768		ENSG00000108061	SHOC2
9864		ENSG00000168264	IRF2BP2
10147		ENSG00000213047	DENND1B
10170		ENSG00000204794	BNIP3L ARHGAP15
10286		ENSG00000165915	SLC39A13
10344		ENSG00000161013	MGAT4B
10356		N/A	MAP2K1IP1
10383		ENSG00000115648	MLPH
10438		ENSG00000120742	SERP1
10483		ENSG00000174106	LEMD3
10516		ENSG00000141198	TOM1L1
10536		ENSG00000204632	HLA-G
10548		N/A	
10631		ENSG00000117154	IGSF21
10657		ENSG00000139832	RAB20
10675		ENSG00000173534	
10682			
10741		ENSG00000136840	ST6GALNAC4
10761		ENSG00000212677	FLJ43870
10776		94239	H2AFV
10825		N/A	
10872		ENSG00000167306	MYO5B
10915		ENSG00000111371	SLC38A1
10952		N/A	
10963		ENSG00000197728	ANK2
10969		ENSG00000122597	
10993		ENSG00000138764	CCNG2
11006			CANX
11011		ENSG00000213957	
11206		729348	BNIP3L
11261		729973	HSPC016
11282		ENSG00000089356	FXYD3
11314		ENSG00000178769	LOC144766 LOC51057
11327		ENSG00000145632	PLK2
11354			CANX
11357		ENSG00000141469	SLC14A1
11439		N/A	
11442		ENSG00000155034	TRPV1
11520		N/A	
11537		ENSG00000009413	LOC144766
11584		3018	HIST1H2BB
11598		ENSG00000204794	
11678			CANX
11722		ENSG00000133195	SLC39A11
11787		ENSG00000186350	RXRA
11826		ENSG00000205231	
11911		ENSG00000203455	
11935		ENSG00000187504	
11948		ENSG00000129103	SUMF2
11963		ENSG00000187555	USP7
12010		ENSG00000092096	SLC22A17
12096		ENSG00000026297	RNASET2
12138			H3F3B
12140		ENSG00000139218	
12151		ENSG00000072736	NFATC3

Table S1 continued

ID	251 list	Gene	Gene Symbol
12216		ENSG00000168036	CTNNB1
12326		729348, 100129791	BNIP3L ARHGAP15
12371		ENSG00000111348	ARHGDIB
12407		ENSG00000063046	EIF4B
12519		N/A	
12602		100132418, 100133130	
12683		ENSG00000080824	HSP90AA1
12858		ENSG00000173867	MRPL46
12991		ENSG00000070756	PABPC1
13016		ENSG00000106603	C7orf44
13029		ENSG00000009413	
13039		ENSG00000132510	JMJD3
13194		ENSG00000180922	
13197			
13199		N/A	
13219		ENSG00000203365	ARHGAP15
13241		ENSG00000100219	XBP1
13269		ENSG00000198125	MB
13359		ENSG00000211451	
13424		ENSG00000197956	S100A6
13464		ENSG00000108946	PRKAR1A
13468		ENSG00000196406	SPANXA2 SPANXC
13477		84820	
13547		ENSG00000114023	C3orf28
13578		ENSG00000091128	LAMB4
13611		ENSG00000178769	LOC51057 EMR4
13616		401602	
13715		ENSG00000113716	KIAA0194
13725		ENSG00000204794	BNIP3L ARHGAP15
13753		ENSG00000175387	SMAD2
13758		ENSG00000164885	CDK5
13848		ENSG00000215492	LOC144983 HNRPA1
13882		ENSG00000117118	SDHB
13940		ENSG00000104824	HNRPL
13986		ENSG00000147854	UHRF2
14003		ENSG00000069431	
14083		ENSG00000057608	GDI2
14235		ENSG00000164040	PGRMC2
14239		ENSG00000147669	POLR2K
14314		727880, 729348	
14414		ENSG00000183072	NKX2-5
14478			CANX
14566		ENSG00000196977	
14579		ENSG00000116209	TMEM59
14584		ENSG00000106541	AGR2
14596		ENSG00000206298	PSMB8
14627		ENSG00000163463	KRTCAP2
14669		ENSG00000196700	KIAA1196
14671		ENSG00000141337	WIPI1
14688		ENSG00000140474	ULK3
14707		ENSG00000170542	SERPINB9
14806		ENSG00000203871	C6orf164
14859		ENSG00000006432	
14904		ENSG00000169253	
15010		N/A	
15043		ENSG00000189058	APOD
15052		ENSG00000140995	FLJ20186
15074		ENSG00000187983	
15157		ENSG00000204525	HLA-B HLA-C
15184		ENSG00000204356	RDBP
15190		ENSG00000163545	NUAK2
15250		ENSG00000214175	HIG2
15263		ENSG00000213007	
15264		N/A	
15500		ENSG00000139697	SBNO1
15552		ENSG00000157778	C7orf48
15607		ENSG00000162006	
15615		ENSG00000130429	ARPC1B
15624		ENSG00000003402	CFLAR
15676		ENSG00000104529	EEF1D
15733		ENSG00000103599	IQCH
15782		ENSG00000161929	
15789		ENSG00000103429	BFAR
15887		ENSG00000163938	GNL3
15943		ENSG00000163884	KLF15
15998		ENSG00000166002	C11orf75
16075		100132418, 100133130	
16092		ENSG00000125999	C20orf114
16277		ENSG00000178769	LOC144766 LOC51057
16309		ENSG00000165175	MID1IP1
16328		ENSG00000101146	
16405		ENSG00000198911	SREBF2
16417		N/A	LOC146110
16488		ENSG00000109519	GRPEL1
16489		23059	CLUAP1
16545		ENSG00000103187	COTL1
16580		ENSG00000177820	FLJ36031
16647		ENSG00000146842	FLJ14803
16848		ENSG00000127266	
16855		ENSG00000072818	CENTB1
16865		N/A	
16867		ENSG00000163083	INHBB
16988		ENSG00000125818	PSMF1
16992		ENSG00000196937	FAM3C
17003		ENSG00000170909	OSCAR
17071		ENSG00000111144	LTA4H
17102		ENSG00000117385	LEPRE1
17103		ENSG00000182196	ARL6IP4
17110		ENSG00000069431	LOC285690
17125		ENSG00000135750	KCNK1
17135		ENSG00000060138	CSDA
17142		ENSG00000180922	MIPOL1 FLJ20298
17209		ENSG00000135525	MAP7
17271		ENSG00000116161	CACYBP
17288		ENSG00000161533	ACOX1
17332		ENSG00000049860	HEXB
17336		N/A	
17365		ENSG00000069431	
17382		ENSG00000120992	LYPLA1
17397		N/A	
17413		ENSG00000103534	TMC5
17422		ENSG00000204794	
17451		ENSG00000171798	KNDC1
17478		ENSG00000213568	
17533		ENSG00000180922	
17559		730092	
17601		ENSG00000180922	LOC51057
17614			H3F3B
17626			H3F3B
17652		ENSG00000160185	UBASH3A
17697		ENSG00000009413	FPGT FLJ35821
17775		ENSG00000112877	CEP72
17778		ENSG00000089053	ANAPC5
17813		ENSG00000175658	
17831		ENSG00000112699	GMDS
17946		ENSG00000084733	RAB10
17982		ENSG00000213007	
18002		ENSG00000084234	APLP2
18076		ENSG00000143001	TMEM61
18204		ENSG00000204794	BNIP3L ARHGAP15
18260		ENSG00000177417	
18291		ENSG00000184612	
18428		ENSG00000204794	
18439		ENSG00000145349	CAMK2D
18474		ENSG00000186502	
18541		ENSG00000204794	BNIP3L ARHGAP15
18578		ENSG00000148444	COMMD3
18637		ENSG00000136682	CBWD1 LOC220869
18692		ENSG00000139977	C14orf35
18710		ENSG00000204794	BNIP3L ARHGAP15
18765		ENSG00000069431	LOC51057
18802		ENSG00000148344	PTGES
18818		ENSG00000211451	
18895		ENSG00000116478	HDAC1
18986		ENSG00000069431	
18992		645694	
19002		ENSG00000135108	FBXO21
19105		N/A	
19148		ENSG00000108576	SLC6A4
19155		ENSG00000009413	
19213		ENSG00000178769	LOC144766 LOC51057
19215		ENSG00000197575	
19242		ENSG00000204794	BNIP3L ARHGAP15
19283		ENSG00000116251	RPL22
19390		ENSG00000149243	FLJ33790
19409		ENSG00000169246	KIAA0220
19413		ENSG00000176105	YES1
19434		ENSG00000180922	
19459		ENSG00000065427	KARS
19490		ENSG00000179823	LOC152217
19494		ENSG00000180922	ODF2L
19514		ENSG00000086619	ERO1LB
19558		ENSG00000149294	NCAM1
19583		ENSG00000203836	LOC376745 LOC200030
19598		ENSG00000085871	MGST2
19619		ENSG00000132849	INADL
19771		ENSG00000175538	TRPV1
19792		ENSG00000108774	RAB5C
19819		ENSG00000114023	C3orf28
19853		ENSG00000176268	

Table S1 continued

ID	251 list	Gene	Gene Symbol
19861		ENSG00000213780	GTF2H4
19891		ENSG00000178769	LOC144766 LOC51057
19913		ENSG00000175895	PLEKHF2
19916		ENSG00000168621	GDNF
20091		ENSG00000143797	MBOAT2
20118		ENSG00000148219	ASTN2
20220		ENSG00000204794	BNIP3L ARHGAP15
20331		ENSG00000178057	C3orf60
20359		ENSG00000196977	
20511		ENSG00000147676	MAL2
20605		ENSG00000102606	ARHGEF7
20621		ENSG00000167645	YIF1B
20674		ENSG00000166226	CCT2
20709		ENSG00000138085	C2orf28
20750			H3F3B
20777		N/A	
20783		ENSG00000171307	ZDHHC16
20790		ENSG00000151276	BAIAP1
20856		ENSG00000106546	AHR
20874			
20959		ENSG00000144452	ABCA12
21083		ENSG00000064666	CNN2
21158		ENSG00000155034	
21279		ENSG00000076554	TPD52
21283		ENSG00000179151	EDC3
21321		728764, 727858	FLJ22795
21357		ENSG00000105619	TFPT
21373		ENSG00000077254	
21474		ENSG00000213705	GNAS
21497		ENSG00000096696	DSP
21632		ENSG00000146282	RARSL
21698		ENSG00000087088	BAX
21735		N/A	ZNF34
21827		ENSG00000143643	TTC13
21837		ENSG00000099219	KIAA1815
21892		ENSG00000104765	BNIP3L
21894			RPS17
21924		ENSG00000002746	HECW1
21980		ENSG00000052344	PRSS8
22004		ENSG00000109861	CTSC
22039		N/A	
22079		ENSG00000183305	MAGEA2
22081		ENSG00000214485	RPL7
22097		ENSG00000176422	SPRYD4
22201		ENSG00000102119	EMD
22203		ENSG00000074800	ENO1
22234		ENSG00000180922	LOC51057
22238		100129434	
22245		ENSG00000189067	LITAF
22255		ENSG00000114648	KLHL18
22296		ENSG00000188428	MUTED
22345		ENSG00000009413	
22352		ENSG00000168385	SEPT2
22464		ENSG00000168374	ARF4
22529		ENSG00000136986	DERL1
22531		ENSG00000112664	NUDT3
22616		ENSG00000111801	BTN3A3
22623		ENSG00000063660	GPC1
22639		ENSG00000071082	
22645		ENSG00000122966	CIT
22711		100131707, 729348	ARHGAP15
22712		ENSG00000215492	
22742		ENSG00000215380	
22743		ENSG00000172292	LASS6
22746		441377, 100131971	
22761		ENSG00000103353	KIAA1970
22788		ENSG00000109332	UBE2D3
22805		ENSG00000156675	RAB11FIP1
22825		ENSG00000204794	BNIP3L ARHGAP15
22855		ENSG00000185862	EVI2B
22910		ENSG00000196977	
22993		ENSG00000069431	LOC285690 DSCR3
22996		ENSG00000206441	HLA-A
23024		257039	
23082		N/A	ANKRD14
23133		ENSG00000175602	CCDC85B
23143		ENSG00000145391	SETD7
23166		ENSG00000180922	
23182		ENSG00000088836	SLC4A11
23227		ENSG00000197152	
23252		ENSG00000168671	UGT3A2
23257		ENSG00000136636	KCTD3
23320		ENSG00000105971	CAV2
23399		N/A	
23400		ENSG00000124256	ZBP1
23481		ENSG00000135535	CD164
23485		ENSG00000122711	SPINK4
23487		ENSG00000111305	GSG1
23514		ENSG00000117335	CD46
23578		ENSG00000213063	
23679		ENSG00000127528	KLF2
23745		ENSG00000177738	SFXN1
23772		ENSG00000032444	PNPLA6
23948		ENSG00000168283	BMI1
23974		ENSG00000215136	FLJ44796
24001		ENSG00000212635	
24011		ENSG00000144381	HSPD1
24013		728620	
24053		ENSG00000214774	NPM1
24074		ENSG00000118007	STAG1
24094		ENSG00000148737	TCF7L2
24112		ENSG00000155034	
24186		ENSG00000117592	PRDX6
24215		ENSG00000111536	IL26
24220		N/A	ZNF195
24337		ENSG00000183524	RPS18
24485		ENSG00000211785	
24492		ENSG00000129116	PALLD
24504		ENSG00000196333	
24537		N/A	
24547		ENSG00000122490	PQLC1
24589		ENSG00000211451	
24592		ENSG00000168658	MGC26733
24630		ENSG00000134548	C12orf39
24656		ENSG00000141424	SLC39A6
24710		ENSG00000150093	ITGB1
24763		ENSG00000164294	
24858		ENSG00000117519	CNN3
24870		ENSG00000180922	FLJ20298 C21orf36
24983		ENSG00000170035	UBE2E3
24996		ENSG00000124214	STAU1
25054		ENSG00000117410	ATP6V0B
25160		ENSG00000143476	DTL
25202		ENSG00000198800	
25217		ENSG00000138193	PLCE1
25233		645694, 727880	
25365		ENSG00000102391	RPL36A
25369		ENSG00000205981	TIM14
25377		ENSG00000180922	
25402		645694, 100129434	
25407		ENSG00000163541	SUCLG1
25441		ENSG00000138078	PREPL
25491		ENSG00000185499	MUC1
25643		ENSG00000113391	C5orf21
25651		ENSG00000204794	BNIP3L ARHGAP15
25757		ENSG00000100266	PACSIN2
25779		ENSG00000161929	
25848		ENSG00000069431	LOC285690 DSCR3
25861		ENSG00000136240	KDELR2
25877		N/A	
25936		ENSG00000214485	RPL7
25956		ENSG00000042753	AP2S1
25963		ENSG00000171995	
25982		ENSG00000091140	DLD
25985		ENSG00000181757	SLC22A18AS
26007		ENSG00000115523	GNLY
26064		ENSG00000108784	NAGLU
26159		N/A	
26180		ENSG00000019102	VSIG2
26236		ENSG00000021355	SERPINB1
26298		ENSG00000173402	DAG1
26371		ENSG00000112110	MRPL18
26568		ENSG00000104518	GSDMDC1
26577		729348, 100129791	BNIP3L ARHGAP15
26624		ENSG00000205268	PDE7A
26701		ENSG00000154760	
26712		ENSG00000054392	HHAT
26716		ENSG00000211451	LOC51057
26783		ENSG00000147677	EIF3S3
26827		ENSG00000178769	
26874		ENSG00000121766	ZCCHC17
26916		ENSG00000169020	ATP5I
26973		N/A	
26985		ENSG00000100196	KDELR3
27050		ENSG00000214271	
27160		ENSG00000013588	GPRC5A
27231		ENSG00000144452	ABCA12
27263		ENSG00000111816	FRK
27274		N/A	
27277		ENSG00000196977	

Table S1 continued

ID	251 list	Gene	Gene Symbol
27333		731049, 27338	
27335		ENSG00000161929	LOC440738
27369		ENSG00000152904	GGPS1
27407		ENSG00000206328	TNF
27421		ENSG00000166974	MAPRE2
27425		ENSG00000152642	GPD1L
27551		ENSG00000076554	TPD52
27566		ENSG00000149658	YTHDF1
27596		ENSG00000141002	TCF25
27600		ENSG00000128739	SNORD107
27676		ENSG00000161929	TRPV1 SCML2
27746		ENSG00000087460	GNAS
27752		ENSG00000184203	PPP1R2
27862		ENSG00000178769	LOC144766 LOC51057
27899		ENSG00000111704	
27932		ENSG00000139722	VPS37B
27947		ENSG00000069431	
28109		ENSG00000099219	KIAA1815
28129		ENSG00000135387	GPIAP1
28192		ENSG00000104723	TUSC3
28220		ENSG00000104529	EEF1D
28300		ENSG00000129625	REEP5
28301		ENSG00000123131	PRDX4
28303		ENSG00000116030	SUMO1
28316		ENSG00000169549	
28325		ENSG00000138802	SEC24B
28342		728678, 727880	
28351		ENSG00000213401	MAGEA3
28426		645694	
28488		ENSG00000160209	PDXK
28549		ENSG00000139631	MEFV NXN
28591		100129434, 100128274	
28603		N/A	
28617		ENSG00000069431	LOC285690 DSCR3
28677		ENSG00000180922	
28708		ENSG00000173366	TLR9
28713		ENSG00000154153	FLJ20152
28723		ENSG00000196586	MYO6
28736		ENSG00000155034	CGNL1
28803		ENSG00000112667	C6orf108
28827		ENSG00000196977	ACOT11
28828		ENSG00000140416	TPM1
28903		ENSG00000080824	
28939		ENSG00000143341	HMCN1
28956		N/A	
28965		ENSG00000152409	JMY
28977		ENSG00000100105	PATZ1
29018		ENSG00000166595	FAM96B
29070		ENSG00000186063	C1orf80
29076		ENSG00000160967	CUTL1
29100		ENSG00000169347	GP2
29230		ENSG00000092470	WDR76
29231		ENSG00000073737	DHRS9
29252		ENSG00000127399	LRRC61
29253		ENSG00000103091	WDR59
29354		ENSG00000165140	FBP1
29406		645694, 727880	
29461		ENSG00000204794	BNIP3L ARHGAP15
29495		ENSG00000163159	VPS72
29505		ENSG00000067225	PKM2
29529		ENSG00000136643	RPS6KC1
29569		ENSG00000178769	LOC144766 LOC51057
29571		ENSG00000212776	
29590		ENSG00000117480	FAAH
29592		ENSG00000105972	C7orf20
29685		727880	
29756		ENSG00000124357	NAGK
29761		ENSG00000099964	MIF
29769		ENSG00000196977	
29848		ENSG00000050628	PTGER3
29909		ENSG00000213526	
29910		ENSG00000204794	BNIP3L ARHGAP15
29925		ENSG00000109586	GALNT7
29929		ENSG00000184779	RPS17
29970		ENSG00000165996	PTPLA
29983		ENSG00000115956	PLEK
30049		ENSG00000189433	GJB4
30075		ENSG00000204525	HLA-C
30103		N/A	
30136		ENSG00000168894	LOC51255
30147		645694, 727880	
30151		ENSG00000114767	RRP9
30236		ENSG00000106305	JTV1
30249		729648	
30303		ENSG00000171124	FUT3
30309		ENSG00000139631	NXN
30319		ENSG00000112893	MAN2A1
30437		ENSG00000137420	
30450		ENSG00000148671	C10orf116
30456		ENSG00000124535	WRNIP1
30507		ENSG00000143321	HDGF
30510			
30517		ENSG00000100129	EIF3S6IP
30555		ENSG00000155034	C10orf46
30601		ENSG00000103316	CRYM
30630			RPS17
30799		ENSG00000213424	KRT222P
30808		ENSG00000206443	
30867		ENSG00000167526	RPL13
30877		ENSG00000100441	KIAA0323
30934		N/A, 6418	SET
30966			RPS17
30984		ENSG00000198265	HELZ
30988		ENSG00000125815	CST8
30998		ENSG00000213872	
31016		ENSG00000125746	EML2
31072		ENSG00000125611	CHCHD5
31112		ENSG00000177045	SIX5
31134		ENSG00000083168	MYST3
31137		ENSG00000181061	HIGD1A
31211		ENSG00000130702	LAMA5
31281		ENSG00000174914	OR9G9
31320		ENSG00000138674	SEC31A
31429		ENSG00000157445	CACNA2D3
31437		ENSG00000123562	MORF4L2
31480		ENSG00000169851	PCDH7
31515		ENSG00000147647	DPYS
31533		ENSG00000176490	DIRAS1
31596		ENSG00000088386	PRKCBP1
31643		ENSG00000179010	MRFAP1
31678		ENSG00000166199	ALKBH3
31703		650405	LOC650405
31716		ENSG00000128438	LOC96597
31763		ENSG00000185499	MUC1
31778		ENSG00000090013	BLVRB
31845		N/A	
31865		ENSG00000178769	LOC51057 EMR4
31869		ENSG00000175809	ZNF645
31934		ENSG00000149257	SERPINH1
32003		ENSG00000065457	ADAT1
32094		22885	ABLIM3
32125		ENSG00000167004	GRP58
32149		ENSG00000215126	CBWD1 LOC220869
32212		ENSG00000160746	TMEM16K
32327		ENSG00000146433	TMEM181
32329		ENSG00000206503	HLA-A
32344		ENSG00000177364	C9orf45
32348		ENSG00000168646	AXIN2
32427		ENSG00000178741	COX5A
32429		ENSG00000204604	
32452		ENSG00000019102	VSIG2
32545		ENSG00000069431	LOC51057
32629		ENSG00000180922	C21orf36
32672		ENSG00000105825	TFPI2
32819		ENSG00000215492	
32872		ENSG00000004059	ARF5
32920		ENSG00000124098	C20orf108
33066		N/A	SORBS1
33201		ENSG00000102241	HTATSF1
33219		ENSG00000174437	ATP2A2
33228		ENSG00000128050	PAICS
33235		ENSG00000075275	CELSR1
33268		ENSG00000213729	
33270		ENSG00000173821	C17orf27
33281		ENSG00000138069	RAB1A
33327		ENSG00000171311	EXOSC1
33329		645694, 644131	
33344		ENSG00000106723	SPIN1
33405		ENSG00000009413	LOC144766 LOC51057
33425		ENSG00000213853	EMP2
33457		ENSG00000101199	ARFGAP1
33463		729348, 100129791	ARHGAP15
33536		ENSG00000125166	GOT2
33540		ENSG00000105576	TNPO2
33555		ENSG00000162877	FLJ32569
33564		ENSG00000124939	SCGB2A1
33586		ENSG00000149091	DGKZ
33605		127545	
33689		ENSG00000144381	
33766			CANX

Table S1 continued

ID	251 list	Gene	Gene Symbol
33833			CANX
33899		ENSG00000125304	TM9SF2
33916		ENSG00000163386	LOC376745 MGC8902
33991		ENSG00000071655	MBD3
34066		ENSG00000135248	NYD-SP18
34093		ENSG00000140832	MARVELD3
34124		ENSG00000125505	LENG4
34234		ENSG00000131386	GALNTL2
34246		ENSG00000069431	
34287		ENSG00000143554	SLC27A3
34289		ENSG00000136436	CALCOCO2
34399		ENSG00000184390	
34407		ENSG00000110628	SLC22A18
34438			CANX
34549		ENSG00000103653	CSK
34569		ENSG00000138867	C22orf13
34647		ENSG00000163867	ZMYM6
34663		ENSG00000079335	CDC14A
34670		ENSG00000138182	ARHGAP15
34698		729348	BNIP3L
34788		ENSG00000170233	NALP1
34836		N/A	
34859		N/A	
34883		ENSG00000114738	MAPKAPK3
34930		ENSG00000196350	
35025		ENSG00000100997	ABHD12
35056		ENSG00000012660	ELOVL5
35058		ENSG00000069431	
35066		ENSG00000165219	GAPVD1
35083		ENSG00000069248	NUP133
35116		ENSG00000148219	
35154		644511	
35182		ENSG00000148985	
35187		ENSG00000198258	UBL5
35193		ENSG00000100056	DGCR14
35259		ENSG00000170275	CRTAP
35270		645694, 727880	
35341		645694, 727880	
35398		ENSG00000174123	TLR10
35404		ENSG00000160838	C1orf92
35470		ENSG00000120937	NPPB
35505		N/A	
35574		ENSG00000163702	IL17RC
35645		ENSG00000213911	
35652		ENSG00000204622	
35759		N/A	
35770			
35781		ENSG00000100503	NIN
35813		ENSG00000213102	
35846		ENSG00000172893	DHCR7
35867		ENSG00000067836	ROGDI
35883		ENSG00000151012	
35889		ENSG00000010256	UQCRC1
35897		1052	CEBPD
35929		ENSG00000174871	CNIH2
35948		ENSG00000104904	OAZ1
35957		ENSG00000213007	
35969		ENSG00000135698	MPHOSPH6
35981		ENSG00000182195	LDOC1
35984		645694	
36031		ENSG00000111319	SCNN1A
36077		ENSG00000157224	CLDN12
36178		ENSG00000184390	TRPV1
36185			RPS17
36197		ENSG00000109606	DHX15
36257		ENSG00000103490	PYCARD
36310		ENSG00000196861	NACAL
36412		ENSG00000160062	ZBTB8
36424		ENSG00000196372	ASB13
36454			RPS17
36567		ENSG00000068878	PSME4
36575		ENSG00000204691	MALAT1
36581		ENSG00000163449	
36601		ENSG00000148219	
36754		ENSG00000133321	RARRES3
36776		ENSG00000198931	APRT
36809		ENSG00000135778	C1orf57
36819		ENSG00000063978	RNF4
36828		ENSG00000198482	ZNF808
36873		ENSG00000103319	EEF2K
36911		ENSG00000172186	
36934		N/A	
36944		ENSG00000160862	AZGP1
37011		ENSG00000115677	HDLBP
37017		N/A	
37080		ENSG00000204632	
37088		ENSG00000151834	GABRA2
37202		ENSG00000215492	HNRPA1
37219		ENSG00000155034	
37221		ENSG00000178814	OPLAH
37223		ENSG00000163113	OTUD7B
37227		ENSG00000121089	
37240		ENSG00000023843	ACCN5
37257		ENSG00000136834	OR1J1
37306		ENSG00000164961	KIAA0196
37445		729348, 100129791	BNIP3L
37463		ENSG00000061918	GUCY1B3
37465		ENSG00000183137	C6orf182
37467		ENSG00000069431	
37477		ENSG00000164951	
37544		ENSG00000104856	RELB
37571		ENSG00000111341	MGP
37579		100131785, 100134537	
37593		ENSG00000135502	SLC26A10
37613		ENSG00000163848	FLJ20195 LOC440738

Table S2. Affymetrix probe set IDs used for hierarchical clustering

**Probe ID**	**Gene Symbol**
205364_at	ACOX2
209173_at	AGR2
202820_at	AHR
216594_x_at	AKR1C1
209699_x_at	AKR1C2
210876_at	ANXA2P1
201097_s_at	ARF4
201288_at	ARHGDIB
218216_x_at	ARL6IP4
218862_at	ASB13
209186_at	ATP2A2
209309_at	AZGP1
203571_s_at	C10orf116
206724_at	CBX4
220565_at	CCR10
204247_s_at	CDK5
213348_at	CDKN1C
203757_s_at	CEACAM6
41660_at	CELSR1
208791_at	CLU
201445_at	CNN3
218048_at	COMMD3
205509_at	CPB1
201380_at	CRTAP
201160_s_at	CSDA
217844_at	CTDSP1
201487_at	CTSC
209366_x_at	CYB5
205417_s_at	DAG1
202428_x_at	DBI
204246_s_at	DCTN3
217973_at	DCXR
219402_s_at	DERL1
201791_s_at	DHCR7
202481_at	DHRS3
218409_s_at	DNAJC1
206782_s_at	DNAJC4
201041_s_at	DUSP1
220942_x_at	E2IG5
210827_s_at	ELF3
201231_s_at	ENO1
221664_s_at	F11R
221856_s_at	FAM63A
209696_at	FBP1
208647_at	FDFT1
218910_at	FLJ10375
219135_s_at	FLJ12681
212995_x_at	FLJ14346
219219_at	FLJ20512
218394_at	FLJ22386
219806_s_at	FN5
214088_s_at	FUT3
202489_s_at	FXYD3
204867_at	GCHFR
221577_x_at	GDF15
201576_s_at	GLB1
215001_s_at	GLUL
214106_s_at	GMDS
205184_at	GNG4
217850_at	GNL3
200708_at	GOT2
202756_s_at	GPC1
212510_at	GPD1L
203108_at	GPRC5A
201106_at	GPX4
212432_at	GRPEL1
201209_at	HDAC1
201944_at	HEXB
217845_x_at	HIG1
214438_at	HLX1
210719_s_at	HMG20B
221791_s_at	HSPC016
208937_s_at	ID1
207826_s_at	ID3
204439_at	IFI44L
209971_x_at	JTV1
200840_at	KARS
204679_at	KCNK1
200700_s_at	KDELR2
204017_at	KDELR3
212356_at	KIAA0323
218342_s_at	KIAA1815
219371_s_at	KLF2
212882_at	KLHL18
203041_s_at	LAMP2
201105_at	LGALS1
208949_s_at	LGALS3 / GALIG
200704_at	LITAF
212449_s_at	LYPLA1
208634_s_at	MACF1
202501_at	MAPRE2
205819_at	MARCO
204179_at	MB
202291_s_at	MGP
204168_at	MGST2
218251_at	MID1IP1
218205_s_at	MKNK2
218211_s_at	MLPH
220615_s_at	MLSTD1
204599_s_at	MRPL28
217165_x_at	MT1F
213693_s_at	MUC1
218231_at	NAGK
204823_at	NAV3
212843_at	NCAM1
207760_s_at	NCOR2
220864_s_at	NDUFA13
206790_s_at	NDUFB1
203190_at	NDUFS8
221567_at	NOL3
205591_at	OLFM1
222025_s_at	OPLAH
201651_s_at	PACSIN2
218019_s_at	PDXK
212094_at	PEG10
204144_s_at	PIGQ
205112_at	PLCE1
201939_at	PLK2
202725_at	POLR2A
209529_at	PPAP2C
200845_s_at	PRDX6
201185_at	PRSS11
202525_at	PRSS8
201053_s_at	PSMF1
210367_s_at	PTGES
221666_s_at	PYCARD
219681_s_at	RAB11FIP1
219622_at	RAB20
201140_s_at	RAB5C
204070_at	RARRES3
217983_s_at	RNASET2
201206_s_at	RRBP1
202449_s_at	RXRA
217728_at	S100A6
204351_at	S100P
203453_at	SCNN1A
200945_s_at	SEC31L1
212268_at	SERPINB1
218675_at	SLC22A17
222217_s_at	SLC27A3
203075_at	SMAD2
202567_at	SNRPD3
217927_at	SPCS1
217995_at	SQRDL
201248_s_at	SREBF2
202506_at	SSFA2
221551_x_at	ST6GALNAC4
201061_s_at	STOM
209154_at	TAX1BP3
209278_s_at	TFPI2
218996_at	TFPT
210130_s_at	TM7SF2
201078_at	TM9SF2
219580_s_at	TMC5
203476_at	TPBG
203786_s_at	TPD52L1
211899_s_at	TRAF4
200972_at	TSPAN3
219481_at	TTC13
213423_x_at	TUSC3
221253_s_at	TXNDC5
218533_s_at	UCKL1
218367_x_at	USP21
212038_s_at	VDAC1
215729_s_at	VGLL1
209216_at	WDR45
203827_at	WIPI49
200670_at	XBP1
212166_at	XPO7
208087_s_at	ZBP1
209494_s_at	ZNF278

Table S3. Characteristics of patients with ERα-positive breast cancer

	Advanced patients	LNN patients
**Surgery**		
Lumpectomy	104	322
Ablation	192	298
**Age at surgery**		
0-40	33	61
41-55	101	219
56-70	108	203
>70	54	137
**Menopausal status at surgery**		
Pre	105	240
Post	191	380
**Adjuvant radiotherapy**		
No	118	253
Yes	178	367
**Tumour size**		
<= 2 cm	81	300
>2cm	215	320
**Nodal status**		
N0	128	620
N1-3	70	0
N>3	98	0
**Metastatic**		
M0	266	620
M1	30	0
**Grade**		
Poor	161	290
Good/moderate	39	130
Unknown	96	200
**Adjuvant systemic therapy**		
None	209	620
Hormonal	0	0
Chemotherapy	56	0
**Progesterone receptor**		
Negative	49	105
Positive	236	477

Table S4. Association of *NCOR2* and *CITED2* with patient and tumour characteristics in 620 ER-positive breast tumours

	N	***NCOR2*** (x10) Med (Iqr)	***CITED2*** Med (Iqr)
**Age (years)** *			
≤40	61	1.43 (1.39)	1.40 (1.09)
41-55	219	1.43 (1.49)	1.52 (1.52)
56-70	203	1.49 (1.40)	2.05 (2.15)
>70	137	1.49 (1.64)	2.25 (2.30)
		NS	*P* = 0.0001
**Menopausal status** #			
Pre	240	1.42 (1.49)	1.48 (1.248)
Post	380	1.48 (1.48)	2.08 (2.09)
		NS	*P* < 0.0001
**Tumour size** #			
≤ 2 cm	300	1.54 (1.72)	1.73 (1.85)
>2 cm	320	1.39 (1.21)	1.79 (1.89)
		NS	NS
**Grade** *			
Poor	290	1.40 (1.37)	1.67 (1.79)
Unknown	200	1.36 (1.46)	1.78 (1.91)
Moderate/good	130	1.84 (1.40)	2.06 (1.81)
		P = 0.0005	*P* = 0.0585

* = *P* for Kruskal-Wallis test
# = *P* for Mann-Whitney U test
Med = Median, Iqr = interquartile range, NS = *P* >0.10

Spearman rank correlations for mRNA levels in 620 ER+ breast tumours

		ESR1	***PGR***	***NCOR2***	***CITED2***
PGR	r_s	0.3273	1		
	P	0.0000			
NCOR2	r_s	0.0688	0.0954	1	
	P	0.0869	0.0176		
CITED2	r_s	0.293	0.1175	0.406	1
	P	0.0000	0.0034	0.0000	
MKI67	r_s	-0.053	-0.189	0.222	-0.050
	P	0.1867	0.0000	0.0000	0.2205
